# TCGA2BED: extracting, extending, integrating, and querying The Cancer Genome Atlas

**DOI:** 10.1186/s12859-016-1419-5

**Published:** 2017-01-03

**Authors:** Fabio Cumbo, Giulia Fiscon, Stefano Ceri, Marco Masseroli, Emanuel Weitschek

**Affiliations:** 1 Institute of Systems Analysis and Computer Science “A. Ruberti”, National Research Council, Via dei Taurini 19, Rome, 00185 Italy; 2 Dipartimento di Elettronica, Informazione e Bioingegneria, Politecnico di Milano, Piazza L. da Vinci 32, Milan, 20133 Italy; 3 Department of Engineering, Roma Tre University, Via della Vasca Navale 79, Rome, 00146 Italy; 4 Department of Engineering, Uninettuno International University, Corso Vittorio Emanuele II 39, Rome, 00186 Italy

**Keywords:** Cancer, Data extraction, Data integration, Knowledge extraction

## Abstract

**Background:**

Data extraction and integration methods are becoming essential to effectively access and take advantage of the huge amounts of heterogeneous genomics and clinical data increasingly available. In this work, we focus on The Cancer Genome Atlas, a comprehensive archive of tumoral data containing the results of high-throughout experiments, mainly Next Generation Sequencing, for more than 30 cancer types.

**Results:**

We propose TCGA2BED a software tool to search and retrieve TCGA data, and convert them in the structured BED format for their seamless use and integration. Additionally, it supports the conversion in CSV, GTF, JSON, and XML standard formats. Furthermore, TCGA2BED extends TCGA data with information extracted from other genomic databases (i.e., NCBI Entrez Gene, HGNC, UCSC, and miRBase). We also provide and maintain an automatically updated data repository with publicly available Copy Number Variation, DNA-methylation, DNA-seq, miRNA-seq, and RNA-seq (V1,V2) experimental data of TCGA converted into the BED format, and their associated clinical and biospecimen meta data in attribute-value text format.

**Conclusions:**

The availability of the valuable TCGA data in BED format reduces the time spent in taking advantage of them: it is possible to efficiently and effectively deal with huge amounts of cancer genomic data integratively, and to search, retrieve and extend them with additional information. The BED format facilitates the investigators allowing several knowledge discovery analyses on all tumor types in TCGA with the final aim of understanding pathological mechanisms and aiding cancer treatments.

**Electronic supplementary material:**

The online version of this article (doi:10.1186/s12859-016-1419-5) contains supplementary material, which is available to authorized users.

## Background

The Cancer Genome Atlas (TCGA) [1] is one of the largest public repositories of genomics, epigenomics, and proteomics data for more than 30 cancer types (http://www.cancergenome.nih.gov/). TCGA includes several Next Generation Sequencing (NGS) [2–5] experimental data types, i.e., Copy Number Variation (CNV) [6], DNA-methylation [7, 8], DNA-sequencing (DNA-seq) [9] including whole genome and whole exome mutations, Gene expression (RNA-seq V1, RNA-seq V2) [10, 11], microRNA sequencing (miRNA-seq) [12], and their meta data (clinical and biospecimen information) [13], which are organized into three levels (1,2,3). In this work, we focus on data extracted from TCGA of level 2 for DNA-seq and of level 3 for the other types of experiments. These are publicly available and high level preprocessed data regarding gene, exon and splice junction expression quantifications, DNA-methylation sites, and genome-wide measurements of DNA mutations and copy number variations. Until June 2016, TCGA experimental and meta data were freely available at TCGA data portal. Recently, most of them have been moved to The Genomic Data Commons (GDC), which is a data sharing platform that promotes precision medicine in oncology (https://gdc.nci.nih.gov/) and where the original TCGA data considered in this work are available [14]. TCGA provides researchers and medical doctors with the largest repository of tumoral and control data collected from thousands of patients, which allows a wide range of analyses for knowledge extraction on several tumor types. However, in order to fully take advantage of this big data repository on cancer, new methods for format standardization, management, integration, and querying of the provided data are required, which aid knowledge discovery for cancer treatment.

Recent works deal with the issue of retrieving, processing, and assembling TCGA data. TCGA-Assembler [15] permits the acquisition, assembling, and processing of public TCGA data and it is based on a collection of R script files mainly used for downloading processed data. The International Cancer Genome Consortium (ICGC) [16] data portal is a comprehensive online archive to characterize genomic abnormalities in several cancer types including data from TCGA. The cBio Cancer Genomics Portal [17] is an open-access resource that provides visualization, analysis, and download of multidimensional cancer genomic data sets based on an R package and a web interface. GeneSpot [18] is a tool designed to view TCGA data from a gene-centric point of view, providing the user with a variety of interactive visualizations of TCGA data. Web-TCGA [19] is an online platform, which focuses on an integrated analysis and visualization of molecular cancer data sets allowing users to generate global molecular profiles across different cancer entities. However, all these tools focus their effort on assembling, analyzing, and visualizing cancer genomic data sets, but do not provide the investigator with a standard and easy accessible format for the TCGA data, which is seamlessly usable for the integration and analysis of these data. Moreover, although also other tools exist to manage and process TCGA data, generally they require programming skills to process the TCGA data, and mainly focus on the analysis and visualization of the most common data types provided by TCGA rather than on providing them in an easy usable standard data format.

In this work, we present the TCGA2BED tool, which implements a procedure to search, retrieve and extend genomic data from TCGA [14], and convert them in the Browser Extensible Data (BED) text format [20]. Besides the BED format, to ensure maximum usage of data, the tool also supports the following set of standard file formats: (i) The Comma Separated Values (CSV) format; (ii) The bioinformatics Gene Transfer Format (GTF) [21]; (iii) The JavaScript Object Notation (JSON) format; (iv) The eXtended Markup Language (XML) format. The rationale behind our choice is the inclusion of standard data storage and bioinformatics formats, which allow us to provide a usable data model for TCGA.

Furthermore, we provide and maintain an automatically updated data repository with publicly available CNV, DNA-methylation, DNA-seq, miRNA-seq, and RNA-seq (V1,V2) experimental data from TCGA converted into the BED format, as well as their meta data in an easy-to-use tab-delimited attribute-value text format. Such formats facilitate the use of these data in knowledge discovery analyses, providing an intuitive and high-quality access to the valuable large-scale cancer genomics and clinical data from TCGA. Moreover, they allow the seamless application on these data of the recently proposed GenoMetric Query Language (GMQL) [22] for their comprehensive management, processing, and querying for knowledge extraction.

## TCGA to BED format definition

According to the TCGA data organization, we refer to each analyzed tissue as a *sample*, and we use as identifier of each genomic experiment present in TCGA the *aliquot*. Such an aliquot is the unit of analysis of TCGA genomic data; it is human readable, tumor-sample specific, and includes the patient, sample, and portion identifiers (patient id, sample id, portion id) followed by two additional fields indicating plate and analysis center (Plate id-Center id), e.g., TCGA-02-0021-01A-01D-0002-04. For further details about TCGA data organization and TCGA barcodes we point the reader to https://wiki.nci.nih.gov/display/TCGA/TCGA+barcode.

For each aliquot, we provide: (i) a *.bed* file, containing the experimental data (i.e., CNV, DNA-methylation, DNA-seq, miRNA-seq, and RNA-seq V1,V2) converted in BED format, and (ii) a *.meta* file, with meta data including clinical and biospecimen data in attribute-value text format; additionally, for each type of data we generate: (iii) a *header.schema* file in EXtended Markup Language (XML) format that describes the structure of the.bed data files, and (iv) a text meta data dictionary file (*metadata_dictionary.txt*) that contains all the distinct meta data attributes with all the values that each attribute assumes in the meta data. Conversely to TCGA, we adopt an aliquot oriented file organization, i.e., we provide a BED file and its associated meta data file for each genomic experiment (identified by an aliquot), in every tumor and experiment type.

The BED format is a column-based format composed of one line per feature (e.g., gene), each including required and optional values for each data column. BED format lines have first three fields required, and nine additional optional fields. Since TCGA data provide more than twelve relevant fields for each data type, we defined a “free” BED format, without limiting the number of additional columns. Hereafter, we refer to our “free” BED definition as BED format. Additionally, we use the *one-based* (one-start or base-counted) genomic coordinate representation, as adopted in the TCGA data. In this coordinate system, the first base of a sequence is one and a region is specified by a closed interval. For instance, the region between the 3^*rd*^ and the 7^*th*^ bases inclusive is [3; 7].

A BED file format includes four specific fields that are found in all the different converted data types; orderly they are:

1. *chrom*, representing the name of the chromosome (e.g., chr3, chrY) where the feature is located
2. *chromStart*, referring to the starting position of the feature in the chromosome or scaffold (according to the one-based coordinate system)
3. *chromEnd*, referring to the ending position of the feature in the chromosome or scaffold (according to the one-based coordinate system)
4. *strand*, defining the DNA strand (either + or –) where the feature is located.

**Fig. 1:**
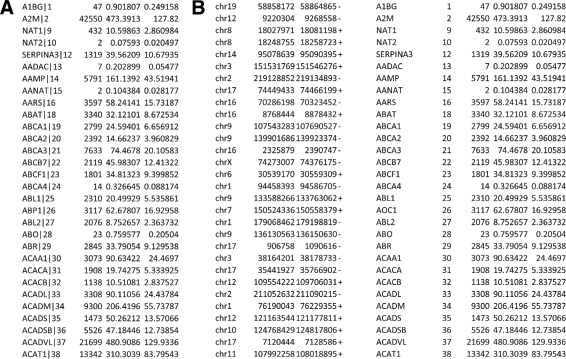
Example of TCGA data belonging to the Kidney Renal Papillary Cell Carcinoma RNA-seq gene quantification experiment. In panel **a** we report the original TCGA data [14] and in panel **b** its converted BED format version (it is worth noting that it has been extended with genomic coordinates)

Then, depending on the analyzed data type and subtype, *N* additional optional fields can be found (Attribute 5, ⋯, Attribute (4+*N*)). The number of the additional fields includes all the attributes of the original TCGA data [14], as well as some *ad-hoc* ones (e.g., in the Spljxn quantification subtype of RNA-seq and RNA-seq V2) which we added to ease the processing and analysis of these data. TCGA2BED enhances data by adding to them annotations (e.g., genomic coordinates) retrieved from well-known repositories, i.e., the NCBI Entrez Gene database (http://www.ncbi.nlm.nih.gov/gene/) [23, 24], the HUGO Gene Nomenclature Committee database (HGNC) (http://www.genenames.org/) [25], the UCSC Genome Browser database (UCSC) (http://genome.ucsc.edu/) [26], and the miRBase (http://www.mirbase.org/) [27] database, and gives as output a BED file. In Fig. 1 panel a we show an example of RNA-seq original TCGA data [14], in panel B its converted and extended representation.

The reader may refer to Additional file 1 for a comprehensive description of the original TCGA and generated file formats for each considered data type. The details about the other supported file formats (CSV, GTF, JSON, and XML) are also reported in Additional file 1 and an example for each format is provided in Additional file 2.

## Implementation

**Fig. 2:**
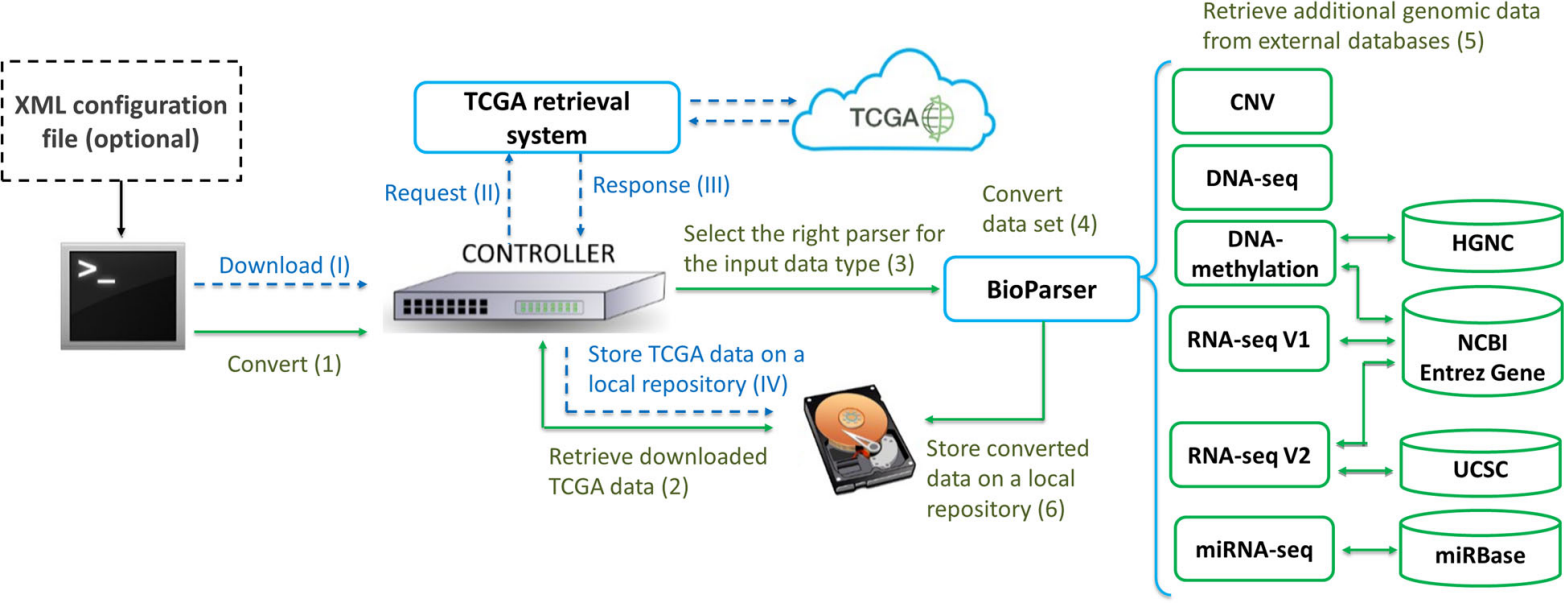
Interaction diagram of the TCGA2BED software architecture. It is composed of: **a** the *controller*, which executes the operations (e.g., download, conversion) specified either with a XML input configuration file or through the user interface; **b** *TCGA retrieval system*, which searches and retrieves TCGA genomic data of multiple types (i.e., CNV, DNA-seq, DNA-methylation, miRNA-seq, and RNA-seq V1, V2) and their associated clinical and biospecimen meta data; **c** the *BioParser*, which converts them in the tab-delimited BED format, and all their corresponding clinical and biospecimen meta data in tab-delimited attribute-value text format. Dashed blue and full green arrowed lines correspond to the paths of data download and conversion, respectively; from left to right, blue thick line rectangles refer to software components, green thin line ones represent the BioParser extensions with the links to the four external databases for additional genomic data retrieval (i.e., UCSC, HGNC, NCBI Entrez Gene, and miRBase). The roman (arabic) numbers refer to the sequence of download (conversion) operations that a user can perform

TCGA2BED is a software tool written in Java programming language that allows extracting, extending, and integrating genomic data as well as associated clinical and biospecimen meta data from TCGA, and transforming them into BED and tab-delimited attribute-value formats, respectively. Additionally, it supports the conversion of the genomic data also in CSV, GTF, JSON, and XML standard formats. The software is available as a desktop application with a simple user interface, and its architecture, which is based on the Model-View-Controller (MVC) pattern [28], follows the flowchart sketched in Fig. 2.

TCGA2BED is composed of three different main components:

- the *Controller*, which either manages user’s commands set through the user interface, or reads and executes an XML input configuration file with the specified operations to be executed;
- the *TCGA retrieval system*, which handles the search and retrieval of public genomic and clinical data available from TCGA [14] by building *ad-hoc* queries;
- the *BioParser*, which converts publicly available TCGA genomic data types (i.e., CNV, DNA-methylation, DNA-seq, miRNA-seq, and RNA-seq V1, V2) into BED format, and all their related clinical and biospecimen meta data into the tab-delimited attribute-value text format. The BioParser is an abstract Java class and it is extended by each parser dedicated to the conversion of each specific data type. Thanks to its abstract nature, the integration of new parser components for other experimental data which would become available is straightforward.

In the flowchart in Fig. 2, dashed blue and full green arrowed lines correspond to two distinct paths: *data download* and *data conversion*, respectively.

For what concerns the *data download*, the dashed blue path starts with an arrow that reaches the Controller component (step I in Fig. 2). It takes as input a list of parameters specifying which type of data or meta data have to be extracted from TCGA, such as the tumor abbreviation tag (e.g., BRCA for Breast Invasive Carcinomar, or OV for Ovarian Carcinoma) and the data type (i.e., CNV, DNA-methylation, DNA-seq, miRNA-seq, and RNA-seq V1, V2). A specific module, through the TCGA retrieval system component, manages the request (step II in Fig. 2) and gets the answer, which includes the location of the requested data (step (III) in Fig. 2). Then, the Controller starts to download the requested data and stores them in a local repository (step IV in Fig. 2).

For what concerns the *data conversion*, the full green path handles the conversion of the genomic data as well as clinical and biospecimen meta data downloaded from TCGA. Again, the first full green arrow reaches the Controller component, specifying the conversion to be performed on the data (step (1) in Fig. 2). Once the data have been retrieved from the local repository, they are ready to be converted (step (2) in Fig. 2). The BioParser component selects the right parser dedicated to the conversion of each specific data type (step (3) in Fig. 2). Then, the conversion procedure (step (4) in Fig. 2) is similar for each data type. As mentioned in the previous Section and in the file formats definition (Additional file 1), not all the information that we include in the n formats are available in the original TCGA data [14] (e.g., gene symbols or genomic coordinates, for some data types). To address this issue, we implemented an information retrieval system (step (5) in Fig. 2) to recover these missing data from some well-known genomic databases, i.e., NCBI Entrez Gene, HGNC, UCSC, and miRBase.

In particular, the NCBI Entrez Gene database [23, 24] is designed to facilitate connections among biological sequences, molecular structures, and scientific papers relevant to specific chromosomal regions. TCGA2BED takes advantage of the REST service of NCBI Entrez system (https://www.ncbi.nlm.nih.gov/books/NBK25501/) to extract from the NCBI Entrez Gene database the genomic coordinates (i.e., chromosome, start, end, and strand) for those genes whose *Entrez Gene id* is the only information provided in the original TCGA data files [14]; specifically, TCGA2BED queries NCBI Entrez for all the gene ids in the gene quantification data subtype of the RNA-seq and RNA-seq V2 data types. Conversely, for what concerns the DNA-methylation data conversion, starting from the *gene symbol*s in the original TCGA data [14], TCGA2BED retrieves the information about the DNA strand where such genes are located (encoded as + or -). For this purpose, TCGA2BED queries another open access database, the HUGO Gene Nomenclature Committee (HGNC) database [25]. It stores and provides all the unique symbols and names for all human loci, to allow unambiguous scientific communication; hence, downloading them locally allows quick extraction of the Entrez Gene id associated with a gene symbol, which in turn TCGA2BED uses to query the NCBI Entrez Gene database to extract the missing information about the DNA strand of the gene. An analogous scenario exists for the *isoform* subtype of the RNA-seq V2 data. In that case, starting from the UCSC *isoform ids* (i.e., transcript ids) in the original TCGA data [14], we obtain first the related Entrez Gene ids, and then all the missing genomic information (i.e., the genomic coordinates). To complete this task, TCGA2BED makes use of the UCSC Genome Browser database [26], that includes the reference sequences and assemblies for a large collection of genomes; TCGA2BED automatically connects to UCSC server and queries it to retrieve the Gene id of each UCSC transcript id, which then uses to query the NCBI Entrez Gene database to extract the missing genomic coordinates. Finally, for the miRNA-seq data conversion, similarly we take advantage of the miRBase database [27], in order to retrieve the genomic coordinates starting from the *miRNA id* available in TCGA. The querying and retrieval processes for all the above-mentioned databases can take long time. Therefore, we periodically track and store locally all the genomic coordinates and Entrez Gene, HGNC, UCSC and miRBase ids to drastically reduce the waiting time for these operations. Lastly, to complete the *data conversion* path, the BioParser stores all the converted data in a local repository (step (6) in Fig. 2).

The TCGA2BED software is available for multiple operating systems, as a Java jar executable with graphic user interface, at http://bioinf.iasi.cnr.it/tcga2bed/. The reader may find installation and usage directions in Additional files 3 and 4.

## Results and discussion

### TCGA2BED data repository

We created and are maintaining an open access FTP repository (ftp://bioinf.iasi.cnr.it/), which contains the original TCGA data sets [14] addressed by TCGA2BED and the corresponding data converted into the BED format.

To increase its usability, the repository is composed of two main folders: *bed* and *tcga_original*.

The *tcga_original* folder contains the original TCGA data files [14] organized in directories, named with the tumor tag of the data, for a total of 33 different tumors. For each tumor the different types of available data are organized in subdirectories, which include also a directory named *meta* with the clinical and biospecimen meta data.

The *bed* folder is composed of 33 directories, each named with a different tumor tag. Each tumor folder includes a set of directories, each for a specific experiment type, containing the publicly available TCGA data of that experiment type for that tumor converted in BED format, and accordingly named, i.e., *cnv*, *dnamethylation*, *dnaseq*, *mirnaseq*, *rnaseq*, *rnaseqv2*. Finally, each experiment folder contains the files in BED format (one for each aliquot), each with the corresponding *.meta* meta data file. For further details about the structure and content of the repository the reader may refer to Additional file 5.

At the time of writing, the final obtained data regarded a total of 33 tumors, 62,335 aliquots, 22,840 samples and 11,317 patients, for a total repository size of 654 GB. It is worth noting that the size of the same experimental and meta data publicly provided by TCGA amounts to 594 GB, which we enhanced with about 60 GB of additional information. The main included tumors are Breast Invasive Carcinoma (BRCA), Kidney Renal Clear Cell Carcinoma (KIRC), Head and Neck Squamous Cell Carcinoma (HNSC), Lung Adenocarcinoma (LUAD), and Brain Lower Grade Glioma (LGG), which count a total of 2,268, 1,096, 1,106, 1,180 and 1,024 samples (tissues), and 1,103, 540, 530, 583, 518 patients, respectively. Table 1 lists the number of included genomic experiments (identified by aliquot ids), samples, and patients for each kind of experiment type across all TCGA tumors; further details are in Additional file 6.

**Table 1:** Number (#) of considered data for each type of experiment, across all TCGA tumors

Experiment type	# Aliquots	# Samples	# Patients	# Tumors
CNV	22,632	22,409	11,162	33
DNA-seq	6,914	6,884	6,852	30
DNA-methylation	12,841	12,508	11,26	33
miRNA-seq	9,909	9,763	9,031	32
RNA-seq V1	3,675	3,674	3,393	15
RNA-seq V2	9,825	9,823	9,107	31
All	62,335	22,840	11,317	33

### Integrative querying of TCGA data with the GenoMetric Query Language

The GenoMetric Query Language [22] is a high-level, declarative query language for genomic data, and a toolkit is available for its use at http://www.bioinformatics.deib.polimi.it/GMQL/. The TCGA data in BED format are fully supported by the GMQL data model and can be seamlessly processed by GMQL, which is a key instrument for the integration of genomic and clinical data also from heterogeneous sources.

**Fig. 3:**
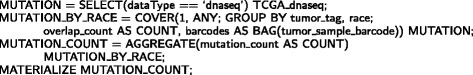
Example of GMQL query on DNA-seq data of TCGA patients that groups samples by tumor type and patient ethnicity, and counts the distinct DNA somatic mutations in each group

Here, we provide three examples of GMQL queries on the TCGA data converted in BED format, which makes straightforward the integration and comprehensive querying of different data types. The first query is reported in Fig. 3, where we take into account DNA-seq data of TCGA patients, group samples by their tumor type and patient ethnicity, and for each ethnic group of every tumor type we extract and count its distinct DNA somatic mutations, counting for each of them the overlaps among the different samples (each sample is identified by its TCGA barcode). It is worth noting that, the COVER operator permits to extract the genomic regions with certain features (e.g., DNA mutations) in the considered samples, and for each extracted region the BAG operator collects the barcodes of the samples with genomic features in that region. Conversely, the AGGREGATE operator counts (through its COUNT aggregate function) the number of distinct mutations in each resulting sample and stores it in the sample metadata; finally, the MATERIALIZE operator returns the obtained result. In particular, with the COVER operator we extract a sample for each tumor type and kind of patient race; the regions in the result samples are non-overlapping and are formed as contiguous intersections of at least one and at most any number of regions (i.e., somatic mutations) in the grouped input samples. For each result region, the COUNT aggregate function in the COVER operator computes the number of feature regions (i.e., mutations) that contribute to create the result region, and the BAG aggregate function gathers the TCGA barcode (identifier) of the sample of each contributing region to keep track of them. The metadata of each final resulting sample are the union of the metadata of the samples in the input data set that regard the same tumor type and patient race, and are enhanced with the number of distinct mutations computed for the tumor type and patient race the sample is referring to.

For example, at the time of writing the number of TCGA DNA-seq data samples regarding the *Kidney Renal Clear Cell Carcinoma* (KIRC) was 235, and the result data set included three samples, one for each ethnic group represented in the KIRC TCGA data; the total numbers of overall DNA somatic mutations in the input samples were 1971, 4913, and 30,940 for the *Asian*, *black or African American*, and *white* race, respectively, and the number of samples for the three races were 7, 20, and 209, respectively, whereas the corresponding numbers of distinct somatic mutations in the result samples were 1049, 1070, and 3227, respectively.

**Fig. 4:**
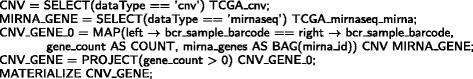
Example of GMQL query on TCGA CNV and miRNA-seq data, which matches samples regarding the same biospecimen and extracts the DNA copy number variations occurring within expressed miRNA genes in the paired samples

In Fig. 4, we report a second GMQL query, which combines Copy Number Variation (CNV) and miRNA-seq data; it searches pairs of TCGA samples of these two data types that regard the same biospecimen, and returns the DNA copy number variations in each CNV sample that are within expressed microRNA (miRNA) genes in the paired miRNA-seq sample. In particular, the MAP operator on CNV and miRNA-seq data sets first joins samples based on the equivalence of their metadata *bcr_sample_barcode* attribute (the identifier for TCGA biospecimens); then, in each pair of samples the COUNT aggregate function calculates the number of miRNA genes overlapping each DNA copy number variation, and the BAG aggregate function collects the miRBase^1^ ids of such genes. Finally, the PROJECT operator selects only those copy number variations of the paired samples that overlap at least one expressed miRNA gene, and the MATERIALIZE operator returns the result. The resulting data set contains only those CNV samples, with their metadata, that have a matching miRNA-seq sample, and containing only their DNA copy number variations (at least one) that occur within an expressed miRNA gene in the matched miRNA-seq sample.

For example, at the time of writing the TCGA CNV and miRNA-seq data samples of *Lung Adenocarcinoma* (LUAD) patients were 1141 and 504, respectively.

The pairs of samples found regarding the same biospecimen were 496; 442 of them contained DNA copy number variations within expressed miRNA genes of the same sample, with an average number of 146 copy number variations per sample.

**Fig. 5:**
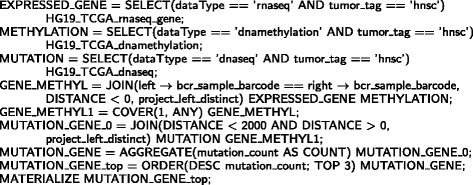
Example of GMQL query on RNA-seq, DNA-methylation and DNA-seq data that finds the DNA somatic mutations within the first 2000 bp outside of the genes both expressed and methylated in at least a TCGA HNSC biospecimen, and extracts these somatic mutations of the top three samples with the highest number of such mutations

Finally, in Fig. 5 we show an example of GMQL query over numerous samples of multiple heterogeneous genomic features from the TCGA repository, seamlessly combined and comprehensively cross-evaluated, together with their clinical and biospecimen metadata, thanks to their availability in BED format and to the data model that GMQL uses. This query applies on RNA-seq, DNA-methylation and DNA-seq data of TCGA *Head and Neck Squamous Cell Carcinoma* (HNSC) patients to find the DNA somatic mutations occurring within the first 2000 bp^2^ outside of the genes that are both expressed and methylated in at least one of these patients, and extracts these mutations of the top three patients with the highest number of such somatic mutations. Specifically, the first JOIN operator applies on RNA-seq gene and DNA-methylation data sets. It first combines samples based on the equivalence of their metadata *bcr_sample_barcode* attribute (the TCGA biospecimen identifier); then, from every pair of samples of each biospecimen it extracts the expressed gene regions that overlap at least a methylation site in the paired DNA methylation sample. Through the COVER operator all these gene regions are then merged in a single sample, which includes the genes both expressed and methylated in at least one of the TCGA HNSC patients. The second JOIN operator applies on this single sample data set and on the entire HNSC DNA-seq data set, and in each sample of the latter one it finds the DNA somatic mutations occurring within the first 2000 bp upstream or downstream of any of the expressed methylated genes extracted. Then, the AGGREGATE operator uses the COUNT aggregate function to determine the number of these mutations in each sample, the ORDER operator ranks the samples according to such number and extracts the top three samples with the highest number of these somatic mutations, and finally the MATERIALIZE operator returns the result.

At the time of writing the RNA-seq, DNA-methylation and DNA-seq samples of TCGA *Head and Neck Squamous Cell Carcinoma* (HNSC) patients were 294, 598, and 279, respectively. Applied on these samples, the described GMQL query found 271 DNA-seq samples with DNA somatic mutations close (within 2000 bp) to the 11,307 genes which were identified as both expressed and methylated. These somatic mutations found in the three samples with the highest number of such mutations were 108, 45, and 36, respectively. The top three samples selected regarded white patients, who were current or current reformed smoker for 15 or less years, with age at the initial pathologic diagnosis of 69, 67 and 68 years, respectively, and presenting 95, 87 and 95% of tumor cells, respectively.

Leveraging on GMQL and TCGA data in BED format, this last example query shows how to easily combine heterogeneous datasets to answer complex biomedical questions, such as to select DNA somatic mutations potentially relevant in altering the regulation of gene expression, which is generally repressed by DNA methylation. Furthermore, thanks to both the availability in easy-to-use tab-delimited attribute-value text format also of the TCGA clinical and biospecimen metadata associated with the genomic data in BED format, as provided by our TCGA2BED software, and their seamless combined processing that GMQL uniquely and innovatively performs, the result dataset (MUTATION_GENE_top) of the query contains also the clinical and biospecimen metadata of the top three samples finally selected. This association between genomic data and their biological/clinical metadata represents one of the new relevant aspects of GMQL, which is not supported by any other system currently available. It allows tracking the provenance of resulting samples and eases the biomedical interpretation of the results, facilitating also result sample stratification and further evaluations.

## Conclusions

TCGA2BED is a software that enables the search, extraction, extension and conversion of The Cancer Genome Atlas genomic data into the BED format, and of their associated clinical and biospecimen meta data in the general tab-delimited attribute-value text format. Additionally, to maximize the usage of the provided data, it supports also the conversion into the CSV, GTF, JSON, and XML general standard formats, allowing the definition of an accessible data model. In TCGA2BED a simple graphical user interface (GUI), as well as a batch interface, are available to search, download and convert publicly available TCGA cancer related data sets; through a user-friendly interface, it is possible to deal with huge amounts of cancer data, and to search, retrieve and extend them with additional information from well-known databases. Additionally, a freely accessible comprehensive FTP server, which contains all public available TCGA CNV, DNA-methylation, DNA-seq, miRNA-seq, and RNA-seq (V1, V2) data converted into the BED format and the related meta data in tab-delimited attribute-value pair format, is released, and periodically updated at ftp://bioinf.iasi.cnr.it/. The availability in BED and in other supported standard formats (i.e., CSV, GTF, JSON, XML) of public genomic TCGA data permits to straightforwardly take full advantage of these very valuable data by reducing the time to be spent in managing them, and allows their seamless integration and comprehensive processing with available bioinformatics tools, such as GMQL. This possibility to globally consider genomic, epigenomic and transcriptomic data of cancer patients, together with their clinical and biospecimen metadata, can give a better view of the patients’ complex biomolecular system, which may lead to novel remarkable findings. In the future, we plan to take advantage of this great opportunity to easily process integratively multiple experimental data types of different kinds of cancer, also from distinct sources, with GMQL, and to perform knowledge extraction analyses on them by combining GMQL with supervised methods [29–31] such as CAMUR [32].

## Availability and requirements

**Project name:** TCGA2BED.**Project home page:** http://bioinf.iasi.cnr.it/tcga2bed.**Operating system(s):** Windows, Linux, and MacOs.**Programming language:** Java.**Other requirements:** Java Runtime Environment (at least version 1.6).**License:** GNU General Public License, version 3 (GPL-3.0).**Any restrictions to use by non-academics:** None.

The FTP repository containing the original TCGA data sets addressed by TCGA2BED and the corresponding data converted into the BED format using TCGA2BED is accessible at ftp://bioinf.iasi.cnr.it/.

## Endnotes

^1^ miRBase (http://www.mirbase.org/) is a database of miRNA sequences and annotations.

^2^ Distances along the DNA are measured in base pairs (bp), i.e., number of nucleotides (or bases) present between two points of the DNA.

## Additional files

Additional file 1 A pdf file that contains all the data format definitions, TCGA to BED format conversion details, and external database integration specifications for each of the considered experiment types. (PDF 1218 kb)

Additional file 2 A compressed archive containing 5 folders that include the example files for each supported format: (i) bed_example; (ii) csv_example; (iii) gtf_example; (iv) json_example; (v) xml_example. (ZIP 37.9 kb)

Additional file 3 A text file that includes installation and execution details of the TCGA2BED software package. (TXT 3.40 kb)

Additional file 4 A pdf file containing the user guide of the TCGA2BED software. (PDF 1085 kb)

Additional file 5 A text file that reports the details about the content and the structure of the TCGA2BED data repository. (TXT 2.56 kb)

Additional file 6 A spreadsheet file containing 3 sheets: (i) *All statistics* includes the patient, sample, and aliquot counts for each tumor and experiment type; (ii) *Counts for each experiment* includes the occurrences of the patients, samples, and aliquots for each experiment (iii) *Total counts for each tumor* includes the occurrences of the patients, samples, and aliquots for each tumor. (XLSX 29.1 kb)

## Abbreviations

API: Application program interface
BED: Browser extensible data
BRCA: Breast invasive carcinoma
CNV: Copy number variation
CSV: Comma separated value
DNA: Deoxyribonucleic acid
FTP: File transfer protocol
GDC: Genomic data commons
GTF: Gene transfer format
GMQL: GenoMetric query language
GUI: Graphical user interface
HGNC: HUGO gene nomenclature committee
HNSC: Head and neck squamous cell carcinoma
HTTP: Hyper text transfer protocol
HUGO: Human genome organisation
ICGC: International cancer genome consortium
ID: Identifier
KIRC: Kidney renal clear cell carcinoma
LGG: Lower grade glioma
LUAD: Lung adenocarcinoma
miRNA: microRNA
NCBI: National Center for Biotechnology Information
REST: REpresentational State transfer
RNA: Ribonucleic acid
RPKM: Reads per kilo base per million mapped reads
SAM: Sequence alignment/map
TCGA: The Cancer Genome Atlas
UCSC: University of California at Santa Cruz
URL: Uniform resource locator
UUID: Universally unique identifier
VCF: Variant call format
XML: eXtensible markup language
