# Supplementary material for: TCGA2BED: extracting, extending, integrating, and querying The Cancer Genome Atlas
Source: BMC Bioinformatics. 2017 Jan 3;18:6. doi: 10.1186/s12859-016-1419-5 (PMC5210259; doi:10.1186/s12859-016-1419-5)
Supplement: Additional file 1 — A pdf file that contains all the data format definitions, TCGA to BED format conversion details, and external database integration specifications for each of the considered experiment types. (PDF 1218 kb) [file 12859_2016_1419_MOESM1_ESM.pdf]

Tool: TCGA2BED			
Web-page: http://bioinf.iasi.cnr.it/tcga2bed/			
Subject: TCGA2BED file format definition			
Document class: Final			
Release: 2.0	*Date:* 14/11/2016	Authors: Emanuel Weitschek, Fabio Cumbo, Giulia Fiscon, Marco Masseroli	**TCGA2BED**

TCGA to BED file format definition

Contents

Introduction 2
DNA Sequencing 4
DNA Methylation 8
RNA-Seq 10
    Gene quantification 10
    Exon quantification 12
    Splice junction (Spljxn) quantification 13
RNA-SeqV2 14
    Exon quantification 14
    Splice junction (Spljxn) quantification 16
    Rsem gene results & Rsem gene normalized results 17
miRNA-Seq 22
Copy Number Variation 25
Clinical and specimen (meta) data 27
Additional output files 29
    Meta data dictionary file 29
    Meta data information files 30
    Experiment information files 30
    Annotations files 30
Additional data file formats 31
    CSV format 31
    XML format 31
    JSON format 32
    GTF format 32

Introduction

Data sets

For the conversion of TCGA data files into the BED format, we actually take into account the following data sets, which include the genomic data that TCGA is currently providing publicly:

- DNA Sequencing (mutations)
- DNA Methylation
- Gene Expression Data (RNA-Seq, RNA-Seq V2 and miRNA-Seq)
- Copy Number Variation - SNP (Single Nucleotide Polymorphism) array-based data
- Clinical and specimen data (metadata)

We use the one-based (one-start or base-counted or fully-closed) genomic coordinate representation, as adopted in the TCGA data files.

We provide the user with all the data sets properly converted in BED format.
In particular, for each data set, the data are provided as follows:

(i) a .tsv **experiment information** file with the number of patients, the number of samples (tissues) and the number of aliquots available for the data set;
(ii) a **.bed** file for each aliquot, containing the **experimental data** converted in standard BED format;
(iii) a **.meta** file for each aliquot, with meta data including the patient **clinical data**;
(iv) a **header.schema** file in xml format that describes the **structure** of the .bed files;
(v) a .txt **metadata dictionary** file that contains all metadata attributes with all the values that each attribute assumes in the metadata

Reference assembly

The genomic coordinates in all converted data sets refer to the human reference assembly GRCh37/hg19.

Data Granularity

We consider the aliquot as data granularity; it is the elementary unit of TCGA, which identifies a single experiment on a tissue. The aliquot is the unit of analysis for TCGA genomic data. Aliquots are the products shipped by the Biospecimen Core Resources to analysis centers. A Biospecimen Core Resource (BCR) is a TCGA center where samples are carefully catalogued, processed, quality-checked and stored along with participant clinical information.
More details are available at:

- https://wiki.nci.nih.gov/display/TCGA/Aliquot
- https://wiki.nci.nih.gov/display/TCGA/TCGA+barcode
- https://wiki.nci.nih.gov/display/TCGA/Biospecimen+Core+Resource

Tumor tags and tumor names

We use the following TCGA tumor tags, which correspond to the following tumor names:

ACC	Adrenocortical carcinoma
BLCA	Bladder Utothelial Carcinoma
BRCA	Breast Invasive Carcinoma
CESC	Cervical squamous cell carcinoma and endocervical adenocarcinoma
CHOL	Cholangiocarcinoma
COAD	Colon adenocarcinoma
DLBC	Lymphoid Neoplasm Diffuse Large B-cell Lymphoma
ESCA	Esophageal carcinoma
GBM	Glioblastoma multiforme
HNSC	Head and Neck squamous cell carcinoma
KICH	Kidney Chromophobe
KIRC	Kidney renal clear cell carcinoma
KIRP	Kidney renal papillary cell carcinoma
LAML	Acute Myeloid Leukemia
LGG	Brain Lower Grade Glioma
LIHC	Liver hepatocellular carcinoma
LUAD	Lung adenocarcinoma
LUSC	Lung squamous cell carcinoma
MESO	Mesothelioma
OV	Ovarian serous cystadenocarcinoma
PAAD	Pancreatic adenocarcinoma
PCPG	Pheochromocytoma and Paraganglioma
PRAD	Prostate adenocarcinoma
READ	Rectum adenocarcinoma
SARC	Sarcoma
SKCM	Skin Cutaneous Melanoma
STAD	Stomach adenocarcinoma
TGCT	Testicular Germ Cell Tumors
THCA	Thyroid carcinoma
THYM	Thymoma
UCEC	Uterine Corpus Endometrial Carcinoma
UCS	Uterine Carcinosarcoma
UVM	Uveal Melanoma

DNA Sequencing

This type of next generation sequencing (NGS) experiment discovers mutations by aligning DNA sequences derived from tumor samples to sequences derived from normal samples and a reference sequence. A MAF file identifies, for each sample, the discovered putative or validated mutations and categorizes those mutations (SNP, deletion, or insertion) as somatic (originating in the tissue) or germline (originating from the germline), as well as the annotation for those mutations.
More details are available at https://wiki.nci.nih.gov/display/TCGA/Mutation+Annotation+Format

Input: one .maf file for each tumor with all DNA-sequencing data is provided by TCGA (refer to Table 1 for field description)

Hugo_Symbol	Entrez_Gene_Id	Center	NCBI_Build	Chromosome	Start_Position	End_Position	Strand	Variant_Classification
A1CF	0	genome.wustl.edu	37	10	52587953	52587953	+	Missense_Mutation
A1CF	0	genome.wustl.edu	37	10	52595854	52595854	+	Missense_Mutation
A1CF	0	genome.wustl.edu	37	10	52595854	52595854	+	Missense_Mutation
A1CF	0	genome.wustl.edu	37	10	52595937	52595937	+	Silent
A1CF	0	genome.wustl.edu	37	10	52596055	52596055	+	Missense_Mutation
A1CF	0	genome.wustl.edu	37	10	52601632	52601632	+	Missense_Mutation
A2M	0	genome.wustl.edu	37	12	9220358	9220359	+	Frame_Shift_Ins
A2M	0	genome.wustl.edu	37	12	9221429	9221429	+	Nonsense_Mutation
A2M	0	genome.wustl.edu	37	12	9230409	9230409	+	Missense_Mutation
A2M	0	genome.wustl.edu	37	12	9242989	9242989	+	Missense_Mutation
A2M	0	genome.wustl.edu	37	12	9242994	9242994	+	Missense_Mutation
A2M	0	genome.wustl.edu	37	12	9246090	9246090	+	Silent
A2M	0	genome.wustl.edu	37	12	9251298	9251298	+	Nonsense_Mutation
A2M	0	genome.wustl.edu	37	12	9254262	9254262	+	Nonsense_Mutation
A2M	0	genome.wustl.edu	37	12	9256962	9256962	+	Missense_Mutation
A2ML1	0	genome.wustl.edu	37	12	8975286	8975286	+	Silent
A2ML1	0	genome.wustl.edu	37	12	8975820	8975820	+	Silent
A2ML1	0	genome.wustl.edu	37	12	8975871	8975871	+	Missense_Mutation
A2ML1	0	genome.wustl.edu	37	12	8988187	8988187	+	Missense_Mutation
A2ML1	0	genome.wustl.edu	37	12	8995897	8995897	+	Silent
A2ML1	0	genome.wustl.edu	37	12	8995942	8995942	+	Silent
A2ML1	0	genome.wustl.edu	37	12	8998092	8998092	+	Nonsense_Mutation
A2ML1	0	genome.wustl.edu	37	12	8998791	8998791	+	Silent
A2ML1	0	genome.wustl.edu	37	12	9001389	9001389	+	Missense_Mutation
A2ML1	0	genome.wustl.edu	37	12	9001389	9001389	+	Missense_Mutation
A2ML1	0	genome.wustl.edu	37	12	9004584	9004584	+	Missense_Mutation
A2ML1	0	genome.wustl.edu	37	12	9004849	9004849	+	Missense_Mutation

BED output format: Tab separated BED file, in which the DNA-seq .maf_file is converted, with the following fields:

1. **chrom** (i.e., the name of the chromosome, e.g., chr3, chrY, chr2_random, retrieved from the 5. field of the TCGA maf file)
2. **chromStart** (i.e., the starting position of the feature in the chromosome or scaffold, e.g., 999, retrieved from the 6. field of the TCGA maf file)
3. **chromEnd** (i.e., the ending position of the feature in the chromosome or scaffold, e.g., 1000, retrieved from the 7. field of the TCGA maf file)
4. **strand** (i.e., it defines the strand, either '+' or '-'., retrieved from the 8. field of the TCGA maf file)
5. **hugo_symbol** (i.e., the symbol of the gene related to the reported variant, if it exists, e.g., “EGFR”, retrieved from the 1. field of the TCGA maf file)
6. **entrez_gene_id** (i.e., the Entrez gene ID of the gene related to the reported variant, if it exists, e.g., “1956”, retrieved from the 2. field of the TCGA maf file)
7. **variant_classification** (i.e., the classification of the reported variant, e.g., “Missense_Mutation”, retrieved from the 9. field of the TCGA maf file)
8. **variant_type** (i.e., the type of mutation, e.g., “INS”, retrieved from the 10. field of the TCGA maf file)
9. **reference_allele** (i.e., the plus strand reference allele at this position, e.g., “A”, retrieved from the 11. field of the TCGA maf file)
10. **tumor_seq_allele1** (i.e., the tumor sequencing (discovery) allele 1, e.g., “C”, retrieved from the 12. field of the TCGA maf file)

11. **tumor_seq_allele2** (i.e., the tumor sequencing (discovery) allele 2, e.g., “G”, retrieved from the 13. field of the TCGA maf file)
12. **dbsnp_rs** (i.e., the latest dbSNP rs ID, e.g., “rs12345”, retrieved from the 14. field of the TCGA maf file)
13. **tumor_sample_barcode** (i.e., the BCR aliquot barcode for the tumor sample, e.g., “TCGA-02-0021-01A-01D-0002-04”, retrieved from the 16. field of the TCGA maf file)
14. **matched_norm_sample_barcode** (i.e., the BCR aliquot barcode for the matched normal sample, e.g., “TCGA-02-0021-10A-01D-0002-04”, retrieved from the 17. field of the TCGA maf file)
15. **match_norm_seq_allele1** (i.e., the Matched normal sequencing allele 1, e.g., “T”, retrieved from the 18. field of the TCGA maf file)
16. **match_norm_seq_allele2** (i.e., the Matched normal sequencing allele 2, e.g., “ACGT”, retrieved from the 19. field of the TCGA maf file)
17. **matched_norm_sample_uuid** (i.e., the BCR aliquot UUID for matched normal, e.g., “567e8487-e29b-32d4-a716-446655443246”, retrieved from the 34. field of the TCGA maf file)

Furthermore:

- Definition of an xml schema that includes the selection of the subset of important attributes and their order by using the same name of the original TCGA attributes; if the attributes is imported as metadata, a special flag in the xml schema is used, e.g., metadata = ”yes”
- The selection is reported in Table 1, where the attributes are highlighted in yellow if imported as region attributes and in green if as metadata.

Notes about TCGA MAF format

- This format is not to be confused with the UCSC Multiple Alignment Format
- It concerns TCGA Level 2 data files
- It regards a tab-delimited file containing only somatic mutations (open access portion of the TCGA Data Portal)
- Mutations are discovered by aligning DNA sequences derived from tumor samples to sequences derived from normal samples and a reference sequence. A MAF file identifies, for each sample, the discovered putative or validated mutations and categorizes those mutations (SNP, deletion, or insertion) as somatic (originating in the tissue) as well as the annotation for those mutations.
- Somatic mutations:
    - Missense and nonsense
    - Splice site, defined as SNP within 2 bp of the splice junction
    - Silent mutations
    - Indels that overlap the coding region or splice site of a gene or the targeted region of a genetic element of interest
    - Frameshift mutations
    - Mutations in regulatory regions
- SNPs:
    - Any germline SNP with validation status "unknown" is included
    - SNPs already validated in dbSNP are not included since they are unlikely to be involved in cancer
- 34 columns are described in Table 1 and are required
- Column headers and values are case sensitive where specified
- Columns may allow null values (i.e. blank cells) and/or have enumerated values

Tool: TCGA2BED			
Web-page: http://bioinf.iasi.cnr.it/tcga2bed/			
Subject: TCGA2BED file format definition			
Document class: Final			
Release: 2.0	*Date:* 14/11/2016	Authors: Emanuel Weitschek, Fabio Cumbo, Giulia Fiscon, Marco Masseroli	**TCGA2BED**

Table 1: Selected fields of the TCGA MAF format

We highlight in yellow the fields which are converted to BED format, in green the fields that are imported as metadata

Id	Header	Description of Values	Example	Enumerated
1	Hugo_Symbol	HUGO symbol for the gene (HUGO symbols are *always* in all caps). If no gene exists within 3kb enter "Unknown". Source: http://genenames.org	EGFR	Set or Unknown
2	Entrez_Gene_Id	Entrez gene ID (an integer). If no gene exists within 3kb enter "0". Source: http://ncbi.nlm.nih.gov/sites/entrez?db=gene	1956	Set
3	Center	Genome sequencing center reporting the variant. If multiple institutions report the same mutation separate list using semicolons. Non-GSC centers will be also supported if center name is an accepted center name.	hgsc.bcm.edu;genome.wustl.edu	Set
4	NCBI_Build	Any TGCA accepted genome identifier. Can be string, integer or a float.	hg18, hg19, GRCh37, GRCh37-lite, 36, 36.1, 37,	Set and Enumerated
5	Chromosome	Chromosome number without "chr" prefix that contains the gene.	X, Y, M, 1, 2, etc.	Set
6	Start_Position	Lowest numeric position of the reported variant on the genomic reference sequence. Mutation start coordinate (1-based coordinate system).	999	Set
7	End_Position	Highest numeric genomic position of the reported variant on the genomic reference sequence. Mutation end coordinate (inclusive, 1-based coordinate system).	1000	Set
8	Strand	Genomic strand of the reported allele. Variants should always be reported on the positive genomic strand. (Currently, only the positive strand is an accepted value).	+	+
9	Variant_Classification	Translational effect of variant allele.	Missense_Mutation	Frame_Shift_Del,Frame_Shift_Ins,In_Frame_Del,In_Frame_Ins,Missense_Mutation, Nonsense_Mutation, Silent,Splice_Site, Translation_Start_Site,Nonstop_Mutation, 3'UTR, 3'Flank, 5'UTR, 5'Flank, IGR[1],Intron,RNA,Targeted_Region
10	Variant_Type	Type of mutation. TNP (tri-nucleotide polymorphism) is analogous to DNP but for 3 consecutive nucleotides. ONP (oligo-nucleotide polymorphism) is analogous to TNP but for consecutive runs of 4 or more.	INS	SNP, DNP, TNP, ONP, INS, DEL, or Consolidated[2]
11	Reference_Allele	The plus strand reference allele at this position. Include the sequence deleted for a deletion, or "-" for an insertion.	A	A,C,G,T and/or -
12	Tumor_Seq_Allele1	Primary data genotype. Tumor sequencing (discovery) allele 1. " -" for a deletion represent a variant. "-" for an insertion represents wild-type allele. Novel inserted sequence for insertion should not include flanking reference bases.	C	A,C,G,T and/or -

13	Tumor_Seq_Allele2	Primary data genotype. Tumor sequencing (discovery) allele 2. " -" for a deletion represents a variant. "-" for an insertion represents wild-type allele. Novel inserted sequence for insertion should not include flanking reference bases.	G	A,C,G,T and/or -
14	dbSNP_RS	Latest dbSNP rs ID (dbSNP_ID) or "novel" if there is no dbSNP record. source: ncbi.nlm.nih.gov/ projects/SNP/	rs12345	Set or "novel"
16	Tumor_Sample_Barcode	BCR aliquot barcode for the tumor sample including the two additional fields indicating plate and well position. i.e. TCGA-SiteID-PatientID-SampleID-PortionID-PlateID-CenterID. The full TCGA Aliquot ID.	TCGA-02-0021-01A-01D-0002-04	Set
17	Matched_Norm_Sample_Barcode	BCR aliquot barcode for the matched normal sample including the two additional fields indicating plate and well position. i.e. TCGA-SiteID-PatientID-SampleID-PortionID-PlateID-CenterID. The full TCGA Aliquot ID; e.g. TCGA-02-0021-10A-01D-0002-04 (compare portion ID '10A' normal sample, to '01A' tumor sample).	TCGA-02-0021-10A-01D-0002-04	Set
18	Match_Norm_Seq_Allele1	Primary data. Matched normal sequencing allele 1. "-" for deletions; novel inserted sequence for INS not including flanking reference bases.	T	A,C,G,T and/or -
19	Match_Norm_Seq_Allele2	Primary data. Matched normal sequencing allele 2. "-" for deletions; novel inserted sequence for INS not including flanking reference bases.	ACGT	A,C,G,T and/or -
28	Sequence_Source	Molecular assay type used to produce the analytes used for sequencing. Allowed values are a subset of the SRA 1.5 library_strategy field values. This subset matches those used at CGHub.	WGS;WXS	Common TCGA values WGS,WGA,WXS,RNA-Seq,miRNA-Seq,Bisulfite-Seq,VALIDATION,Other,Other allowed values (per SRA 1.5),ncRNA-Seq,WCS,CLONE,POOLCLONE,AMPLICON,CLONEEND,FINISHING,ChIP-Seq,MNase-Seq,DNase-Hypersensitivity,EST,FL-cDNA,CTS,MRE-Seq,MeDIP-Seq,MBD-Seq,Tn-Seq,FAIRE-seq,SELEX,RIP-Seq,ChIA-PET
32	Sequencer	Instrument used to produce primary data. Separate multiple entries using semicolons.	Illumina GAIIx;SOLID	Illumina GAIIx<br>Illumina HiSeq<br>SOLID454<br>ABI 3730xl<br>Ion Torrent PGM<br>Ion TorrentProton<br>PacBio RS<br>Illumina MiSeq<br>Illumina HiSeq2500<br>454 GS FLX Titanium<br>AB SOLiD 4 System
33	Tumor_Sample_UUID	BCR aliquot UUID for tumor sample	550e8400-e29b-41d4-a716-446655440000	
34	Matched_Norm_Sample_UUID	BCR aliquot UUID for matched normal	567e8487-e29b-32d4-a716-446655443246	

DNA Methylation

Another wide-spread NGS experiment is large-scale analysis of DNA methylation, which consists in deep sequencing of bisulfite-treated DNA. DNA methylation can be defined as the covalent modification of cytosine bases at the C-5 position, generally within a CpG sequence context. If DNA methylation occurs in promoter regions, it is an epigenetic mark that represents the inactivity of the transcripts.
More details are available at https://wiki.nci.nih.gov/display/TCGA/DNA+methylation.
We consider both the HumanMethylation27 and HumanMethylation450 DNA methylation platforms.

Input:
One tab delimited file is provided by TCGA for each aliquot, with the following fields:

1. Composite Element REF (i.e., the composite element reference is used to record the location of what is aligned to (hg19 assembly))
2. Beta_value (i.e., the beta-value is the ratio of the methylated probe intensity and the overall intensity (i.e., sum of methylated and un-methylated probe intensities))
3. Gene_Symbol (i.e., the symbol of the gene where the methylation occurs)
4. Chromosome (i.e., the chromosome where the methylation occurs)
5. Genomic_Coordinate (i.e., the genomic coordinates of the probed CpG dinucleotide (a CpG island is where a cytosine nucleotide occurs next to a guanine nucleotide))

Each row in the input file refers to a single CpG island.

Hybridization REF	TCGA-AR-A1AH-01A-11D-A12E-05	TCGA-AR-A1AH-01A-11D-A12E-05	TCGA-AR-A1AH-01A-11D-A12E-05	TCGA-AR-A1AH-01A-11D-A12E-05
Composite Element REF	Beta_value	Gene_Symbol	Chromosome	Genomic_Coordinate
cg00000292	0.834741168636629	ATP2A1	16	28890100
cg00002426	0.0744079229874152	SLMAP	3	57743543
cg00003994	0.0556194629813211	MEOX2	7	15725862
cg00005847	0.87528775457464	HOXD3	2	177029073
cg00006414	NA	ZNF425;ZNF398	7	148822837
cg00007981	0.0383908291103455	PANX1	11	93862594
cg00008493	0.984646115838741	COX8C;KIAA1409	14	93813777
cg00008713	0.0196319352920381	IMPA2	18	11980953
cg00009407	0.0194265363203796	TTC8	14	89290921
cg00010193	0.528833478864518	NA	NA	0
cg00011459	0.935923629534861	TMEM186;PMM2	16	8890425
cg00012199	0.0166996279263634	ANG;RNASE4	14	21151024
cg00012386	0.0128372066733585	JMJD4;SNAP47	1	227922512
cg00012792	0.0222726748535999	MUTED	6	8064493
cg00014085	0.0174606060437858	ELMOD3;RETSAT	2	85581505
cg00014837	NA	ACRBP	12	6757257
cg00015770	0.735244049702383	QRFPR	4	122302007
cg00016968	0.237478073283552	RHOC	1	113250448
cg00019495	0.10017917014331	HOPX	4	57547525
cg00020533	0.945287498955943	TULP1	6	35480916
cg00021527	0.0119511131148166	TAF15	17	34136180
cg00022606	NA	TBC1D20	20	442445
cg00022866	0.785819243962471	CCDC88B	11	64108440
cg00024396	0.0548181202391892	ELOVL5	6	53214008
cg00024812	0.0169161074275637	ITGB1BP1;CPSF3	2	9564248
cg00025138	0.0121598316286783	MAP3K9	14	71275917
cg00025991	0.589397143179557	DIP2C	10	736625

BED output format: Tab separated BED file, in which the DNA Methylation_file is converted, with the following fields:

1. **chrom** (retrieved from the 4. field of the TCGA DNA methylation file, e.g., "16")
2. **chromStart** (retrieved from the 5. field of the TCGA DNA methylation file, e.g., 28890100)
3. **chromEnd** (equal to chromStart, since methylation involve a single base and the used genomic coordinate system is 1-based)
4. **strand** (retrieved from NCBI Entrez Gene database[1], based on the Entrez Gene ID retrieved from HUGO Gene Nomenclature Committee (HGNC)[2] according to the human gene symbol provided in field 7, e.g., '+')
5. **composite_element_ref** (retrieved from the 1. field of the TCGA DNA methylation file, e.g., "cg00000292")
6. **beta_value** (retrieved from the 2. field of the TCGA DNA methylation file, e.g., 0.834741168636629)
7. **gene_symbol** (retrieved from the 3. field of the TCGA DNA methylation file, e.g., "ATP2A1")

Note that:

- Missing values of attributes are labelled with the string "null"
- It is worth noting that, in TCGA, DNA methylation refers to the covalent modification of cytosine bases at the C-5 position, generally within a CpG sequence context.

[1] All the NCBI queries are performed according to the following rest query and by taking into account the GRCh37 (hg19) reference genome: http://eutils.ncbi.nlm.nih.gov/entrez/eutils/efetch.fcgi/?db=Gene&id=ID
[2] Queries to HUGO Gene Nomenclature Committee (HGNC):are performed according to the following rest query http://rest.genenames.org/fetch/symbol/ followed by gene symbol, e.g., http://rest.genenames.org/fetch/symbol/BRCA1

RNA-Seq

RNA-Seq data contain information about both nucleotide sequence and gene expression that is quantified by using the RPKM (Reads Per Kilobase of exon model per Million mapped reads) method.

Three files are provided by TCGA for each aliquot:

- Gene quantification (i.e., the calculated expression signal of a gene)
- Exon quantification (i.e., the calculated expression signal of a particular composite exon of a gene)
- Splice junction (Spljxn) quantification (i.e., the calculated expression signal of a particular composite splice junction of a gene)

More details are available at https://wiki.nci.nih.gov/display/TCGA/RNASeq

Gene quantification

Input:

One tab delimited file is provided by TCGA for each aliquot, with the following fields:

1. Gene (i.e., the gene whose expression is quantified)
2. Raw_counts (i.e., the sum of fractions of reads (rounded off to the nearest integer - restricted by the RNA-seq validator) that mapped to collapsed transcripts representing a specific gene)
3. Median_length_normalized (i.e., the average coverage over all exons in the collapsed transcripts, defined as the sum of the coverage depths at each base in all exons divided by the sum of the exon lengths)
4. RPKM (i.e., Reads Per Kilobase per Million mapped reads, see Mortazavi et al., Nat Methods 2008, for the detailed explanation)

Each row in the input file refers to a single gene.

<table>
<tr><th>gene</th><th>raw_counts</th><th>median_length_normalized</th><th>RPKM</th></tr>
<tr><td>?|100130426</td><td>0</td><td>0</td><td>0</td></tr>
<tr><td>?|100133144</td><td>112</td><td>4.56043046357616</td><td>1.30933474307884</td></tr>
<tr><td>?|100134869</td><td>76</td><td>2.38393977415307</td><td>0.673325215610896</td></tr>
<tr><td>?|10357</td><td>197</td><td>15.4827044025157</td><td>4.37430150520734</td></tr>
<tr><td>?|10431</td><td>3291</td><td>147.160107334526</td><td>41.5705415640392</td></tr>
<tr><td>A1BG|1</td><td>514</td><td>9.99567949725059</td><td>2.8510466487858</td></tr>
<tr><td>A1CF|29974</td><td>1</td><td>0.0231588698471515</td><td>0.0065410423544268</td></tr>
<tr><td>A2BP1|54715</td><td>0</td><td>0</td><td>0</td></tr>
<tr><td>A2LD1|87769</td><td>194</td><td>7.61811023622047</td><td>2.15723576848996</td></tr>
<tr><td>A2ML1|144568</td><td>80</td><td>1.09807162534435</td><td>0.311231084147823</td></tr>
<tr><td>A2M|2</td><td>19459</td><td>216.476519029602</td><td>61.1622851356274</td></tr>
<tr><td>A4GALT|53947</td><td>245</td><td>5.775</td><td>1.63203634838954</td></tr>
<tr><td>A4GNT|51146</td><td>0</td><td>0</td><td>0</td></tr>
<tr><td>AAA1|404744</td><td>0</td><td>0</td><td>0</td></tr>
<tr><td>AAAS|8086</td><td>2566</td><td>71.402561247216</td><td>20.176690087567</td></tr>
<tr><td>AACSL|729522</td><td>2</td><td>0.035486160397445</td><td>0.0100227895267619</td></tr>
<tr><td>AACS|65985</td><td>3905</td><td>62.5841615902533</td><td>17.6809366081196</td></tr>
<tr><td>AADACL2|344752</td><td>0</td><td>0</td><td>0</td></tr>
<tr><td>AADACL3|126767</td><td>0</td><td>0</td><td>0</td></tr>
<tr><td>AADACL4|343066</td><td>0</td><td>0</td><td>0</td></tr>
<tr><td>AADAC|13</td><td>8</td><td>0.231884057971014</td><td>0.0654938455337158</td></tr>
</table>

BED output format: Tab separated BED file, in which the RNA-seq Gene quantification file is converted, with the following fields:

1. **chrom** (retrieved from NCBI Entrez Gene database[3], according to the entrez gene id provided in field 6, e.g., “chr4”)
2. **chromStart** (retrieved from NCBI Entrez Gene database[3], according to the entrez gene id provided in field 6, e.g., 109740692)
3. **chromEnd** (retrieved from NCBI Entrez Gene database[3], according to the entrez gene id provided in field 6, e.g., 109802225)
4. **strand** (retrieved from NCBI Entrez Gene database[3], according to the entrez gene id provided in field 6, e.g., ‘-’)
5. **gene_symbol** (retrieved from the 1. field of the TCGA RNA-Seq file, part before “|”, e.g., “CFI”)
6. **entrez_gene_id** (retrieved from the 1. field of the TCGA RNA-Seq file, part after “|”, e.g., “3426”)
7. **raw_counts** (retrieved from the 2. field of the TCGA RNA-Seq file, e.g., 2032)
8. **median_length_normalized** (retrieved from the 3. field of the TCGA RNA-Seq file, e.g., 46.6146072576941)
9. **rpkm** (retrieved from the 4. field of the TCGA RNA-Seq file, e.g., 8.95924060998011)

[3] All the NCBI queries are performed according to the following rest query and by taking into account the GRCh37 (hg19) reference genome: http://eutils.ncbi.nlm.nih.gov/entrez/eutils/efetch.fcgi/?db=Gene&id=ID

Exon quantification

Input:
One tab delimited file is provided by TCGA for each aliquot, with the following fields:

1. Exon (i.e., the exon whose expression is quantified)
2. Raw_counts (i.e., the sum of fractions of reads that mapped to an exon)
3. Median_length_normalized (i.e., the average coverage over the exon, defined as the sum of the coverage depths at each base in an exon divided by the length of the exon)
4. RPKM (i.e., Reads Per Kilobase per Million mapped reads, see Mortazavi et Al, Nat Methods 2008, for the detailed explanation)

Each row in the input file refers to a single exon.

exon	raw_counts	median_length_normalized	RPKM
chr1:11874-12227:+	150	0.423728813559322	0.11967890206108
chr1:12595-12721:+	0	0	0
chr1:12613-12721:+	0	0	0
chr1:12646-12697:+	0	0	0
chr1:13221-14409:+	427	0.359125315391085	0.101432147338092
chr1:13403-14409:+	427	0.4240317775571	0.119764471881819
chr1:16765-14363:-	53072	22.0857261756138	6.23794128540913
chr1:17055-16854:-	8944	44.2772277227723	12.505758004008
chr1:18061-17233:-	32337	39.0072376357057	11.0172903595175
chr1:18379-18268:-	6521	58.2232142857143	16.4446932500278
chr1:18554-18497:-	500	8.62068965517241	2.43484662813922
chr1:19759-18913:-	12734	15.0342384887839	4.24630352736255
chr1:24901-24738:-	8961	54.640243902439	15.4327111806807
chr1:29370-29321:-	987	19.74	5.57540920297831
chr1:29961-29824:-	0	0	0
chr1:35174-34612:-	0	0	0
chr1:35481-35277:-	901	4.39512195121951	1.24136795212975
chr1:36081-35721:-	0	0	0
chr1:69091-70008:+	0	0	0
chr1:90404-89295:-	21657	19.5108108108108	5.51067650213593
chr1:139228-137839:-	15164	10.9093525179856	3.08126162245753

BED output format: Tab separated BED file, in which the RNA-seq Exon quantification file is converted, with the following fields:

1. **chrom** (retrieved from the 1. field of the TCGA RNA-Seq file, part before the first ":", e.g., "chr1")
2. **chromStart** (retrieved from the 1. field of the TCGA RNA-Seq file, part just after the first ":", e.g., 11874)
3. **chromEnd** (retrieved from the 1. field of the TCGA RNA-Seq file, part just before the second ":", e.g.,12227)
4. **strand** (retrieved from the 1. field of the TCGA RNA-Seq file, part just after the second ":", e.g., '+')
5. **raw_counts** (retrieved from the 2. field of the TCGA RNA-Seq file, e.g., 150)
6. **median_length_normalized** (retrieved from the 3. field of the TCGA RNA-Seq file, e.g., 0.423728813559322)
7. **rpkm** (retrieved from the 4. field of the TCGA RNA-Seq file, e.g., 0.11967890206108)

Splice junction (Spljxn) quantification

Input:
One tab delimited file is provided by TCGA for each aliquot, with the following fields:

1. Junction (i.e., the coordinates of an exon-exon junction)
2. Raw_counts (i.e., the sum of fractions of reads that mapped to exon-exon junctions)

Each row in the input file refers to a single splice junction.

junction	raw_counts
chr1:12227:+,chr1:12595:+	0
chr1:12227:+,chr1:12613:+	0
chr1:12227:+,chr1:12646:+	0
chr1:679575:-,chr1:678730:-	7
chr1:701709:-,chr1:700627:-	25
chr1:703928:-,chr1:701767:-	16
chr1:704877:-,chr1:703993:-	22
chr1:708356:-,chr1:705092:-	19
chr1:709551:-,chr1:708487:-	22
chr1:713664:-,chr1:709660:-	12
chr1:763155:+,chr1:764383:+	8
chr1:764484:+,chr1:783034:+	5
chr1:764484:+,chr1:787307:+	1
chr1:764484:+,chr1:788051:+	0
chr1:783186:+,chr1:787307:+	1
chr1:787490:+,chr1:788051:+	7
chr1:787490:+,chr1:788771:+	3
chr1:788146:+,chr1:788771:+	10
chr1:788902:+,chr1:788957:+	1
chr1:809492:-,chr1:804055:-	0
chr1:812126:-,chr1:810535:-	1

BED output format: Tab separated BED file, in which the RNA-seq Spljxn quantification file is converted, with the following fields:

1. **chrom** (retrieved from the 1. field of the TCGA RNA-Seq file, part just before the first ":", e.g., "chr1")
2. **chromStart** (retrieved from the 1. field of the TCGA RNA-Seq file, part just after the first ":", e.g., 12227)
3. **chromEnd** (retrieved from the 1. field of the TCGA RNA-Seq file, part just after the third ":", e.g., 12595)
4. **strand** (retrieved from the 1. field of the TCGA RNA-Seq file, part just after the second ":", e.g., '+')
5. **raw_counts** (retrieved from the 2. field of the TCGA RNA-Seq file, e.g., 0)
6. **inner_left** = chromStart + 1
7. **inner_right** = chromEnd - 1

It is worth noting that, each junction is defined by the last position of exon N and the first position of exon N+1.
Quantification at the splice junction level is also calculated based on the aligned reads converted to genomic coordinates. The only reported value for this level is raw read counts, which is determined by the number of reads that cross a particular junction. Because a splice junction has an effective length of zero, the coverage and RPKM calculations do not apply.
Since in 1-based coordinate system it is not possible to represent regions of length zero, we add two additional fields called inner_left = chromStart + 1 e inner_right = chromEnd - 1 in order to have the intronic region location.

RNA-SeqV2

RNA-Seq Version 2 is similar to RNA-Seq in that it uses sequencing data to determine gene expression levels. RNA-Seq V2 experimental files contain data obtained with a different normalization technique than in RNA-Seq files, which is based on RSEM (RNA-Seq by Expectation Maximization) described in *Bo L, Dewey CN. RSEM: accurate transcript quantification from RNA-Seq data with or without a reference genome. BMC Bioinformatics 2011;12: 323*.

Six files are provided by TCGA for each aliquot:

- Exon quantification (i.e., the calculated expression signal of a particular composite exon of a gene)
- Splice junction (spljxn) quantification (i.e., the calculated expression signal of a particular composite splice junction of a gene)
- Rsem gene results (i.e., the raw expression signal for the expression of a gene)
- Rsem gene normalized results (i.e., the normalized results for the expression of a gene)
- Rsem isoform results (i.e., the raw expression signal of individual isoforms (transcripts))
- Rsem isoform normalized results (i.e., the normalized expression signal of individual isoforms (transcripts))

More details are available at https://wiki.nci.nih.gov/display/TCGA/RNASeq+Version+2

Exon quantification

Input:
One tab delimited file is provided by TCGA for each aliquot, with the following fields:

1. Exon (i.e., the exon whose expression is quantified)
2. Raw_counts (i.e., the sum of fractions of reads that mapped to an exon)
3. Median_length_normalized (i.e., the average coverage over the exon, defined as the sum of the coverage depths at each base in an exon divided by the length of the exon)
4. RPKM (i.e., Reads Per Kilobase per Million mapped reads, see Mortazavi et al., Nat Methods 2008, for the detailed explanation)

Each row in the input file refers to a single exon.

exon	raw_counts	median_length_normalized	RPKM
chr1:11874-12227:+	150	0.423728813559322	0.11967890206108
chr1:12595-12721:+	0	0	0
chr1:12613-12721:+	0	0	0
chr1:12646-12697:+	0	0	0
chr1:13221-14409:+	427	0.359125315391085	0.101432147338092
chr1:13403-14409:+	427	0.4240317775571	0.119764471881819
chr1:16765-14363:-	53072	22.0857261756138	6.23794128540913
chr1:17055-16854:-	8944	44.2772277227723	12.5057580004008
chr1:18061-17233:-	32337	39.0072376357057	11.0172903595175
chr1:18379-18268:-	6521	58.2232142857143	16.4446932500278
chr1:18554-18497:-	500	8.62068965517241	2.43484662813922
chr1:19759-18913:-	12734	15.0342384887839	4.24630352736255
chr1:24901-24738:-	8961	54.640243902439	15.4327111806807
chr1:29370-29321:-	987	19.74	5.57540920297831
chr1:29961-29824:-	0	0	0
chr1:35174-34612:-	0	0	0
chr1:35481-35277:-	901	4.39512195121951	1.24136795212975
chr1:36081-35721:-	0	0	0
chr1:69091-70008:+	0	0	0
chr1:90404-89295:-	21657	19.5108108108108	5.51067650213593
chr1:139228-137839:-	15164	10.9093525179856	3.08126162245753

BED output format: *Tab separated BED file*, in which the RNA-seq V2 Exon quantification file is converted, with the following fields:

1. **chrom** (retrieved from the 1. field of the TCGA RNA-Seq V2 file, part just before the first ":", e.g., "chr1")
2. **chromStart** (retrieved from the 1. field of the TCGA RNA-Seq V2 file, part just after the first ":", e.g., 11874)
3. **chromEnd** (retrieved from the 1. field of the TCGA RNA-Seq V2 file, part just before the second ":", e.g., 12227)
4. **strand** (retrieved from the 1. field of the TCGA RNA-Seq V2 file, part just after the second ":", e.g., '+')
5. **raw_counts** (retrieved from the 2. field of the TCGA RNA-Seq V2 file, e.g., 150)
6. **median _length_normalized** (retrieved from the 3. field of the TCGA RNA-Seq V2 file, e.g., 0.423728813559322)
7. **rpkm** (retrieved from the 4. field of the TCGA RNA-Seq V2 file, e.g., 0.11967890206108)

Splice junction (Spljxn) quantification

Input:
One tab delimited file is provided by TCGA for each aliquot, with the following fields:

1. Junction (i.e., the coordinates of an exon-exon junction)
2. Raw_counts (i.e., the sum of fractions of reads that mapped to exon-exon junctions)

Each row in the input file refers to a single splice junction.

junction	raw_counts
chr1:12227:+,chr1:12595:+	0
chr1:12227:+,chr1:12613:+	0
chr1:12227:+,chr1:12646:+	0
chr1:679575:-,chr1:678730:-	7
chr1:701709:-,chr1:700627:-	25
chr1:703928:-,chr1:701767:-	16
chr1:704877:-,chr1:703993:-	22
chr1:708356:-,chr1:705092:-	19
chr1:709551:-,chr1:708487:-	22
chr1:713664:-,chr1:709660:-	12
chr1:763155:+,chr1:764383:+	8
chr1:764484:+,chr1:783034:+	5
chr1:764484:+,chr1:787307:+	1
chr1:764484:+,chr1:788051:+	0
chr1:783186:+,chr1:787307:+	1
chr1:787490:+,chr1:788051:+	7
chr1:787490:+,chr1:788771:+	3
chr1:788146:+,chr1:788771:+	10
chr1:788902:+,chr1:788957:+	1
chr1:809492:-,chr1:804055:-	0
chr1:812126:-,chr1:810535:-	1

BED output format: Tab separated BED file, in which the RNA-seq V2 Spljxn quantification file is converted, with the following fields:

1. **chrom** (retrieved from the 1. field of the TCGA RNA-Seq V2 file, part just before the first ":", e.g., "chr1")
2. **chromStart** (retrieved from the 1. field of the TCGA RNA-Seq V2 file, part just after the first ":", e.g., 12227)
3. **chromEnd** (retrieved from the 1. field of the TCGA RNA-Seq file V2, part just before the third ":", e.g., 12595)
4. **strand** (retrieved from the 1. field of the TCGA RNA-Seq V2 file, part just after the second ":",, e.g., '+')
5. **raw_counts** (retrieved from the 2. field of the TCGA RNA-Seq V2 file, e.g., 0)
6. **inner_left** = chromStart + 1
7. **inner_right** = chromEnd - 1

Rsem gene results & Rsem gene normalized results

Input:
One tab delimited file is provided by TCGA for each aliquot, with the following fields:

1. Gene_id (i.e., the Entrez gene ID of the gene whose expression is quantified)
2. Raw_count(*) (i.e., see “Important remarks” on page 20)
3. Scaled_estimate(*) (i.e., see “Important remarks” on page 20)
4. Transcript_id (i.e., the UCSC database ID of the transcript)

Each row in the input file refers to a single gene.

Input normalized:
Another tab delimited file is provided by TCGA for each aliquot, with the following fields:

1. Gene_id (i.e., the Entrez gene ID of the gene whose expression is quantified)
2. Normalized_count (i.e., RSEM expression estimates normalized to set the upper quartile count at 1000 for gene level estimates, by dividing all "raw_count" values by the 75th percentile of the raw counts (after removing zeros) and multiply that by 1000[4])

Each row in the input file refers to a single gene.

We merge the two original TCGA files in one single BED file by adding the normalized_count field after the other fields.

<table>
<tr><th>gene_id</th><th>raw_count</th><th>scaled_estimate</th><th>transcript_id</th></tr>
<tr><td>?|100130426</td><td>0</td><td>0</td><td>uc011lsn.1</td></tr>
<tr><td>?|100133144</td><td>17.32</td><td>1.36E-06</td><td>uc010unu.1,uc010uoa.1</td></tr>
<tr><td>?|100134869</td><td>46.68</td><td>2.70E-06</td><td>uc002bgz.2,uc002bic.2</td></tr>
<tr><td>?|10357</td><td>4</td><td>6.85E-07</td><td>uc010zzl.1</td></tr>
<tr><td>?|10431</td><td>882</td><td>7.23E-05</td><td>uc001jiu.2,uc010qhg.1</td></tr>
<tr><td>?|136542</td><td>0</td><td>0</td><td>uc011krn.1</td></tr>
<tr><td>?|155060</td><td>502</td><td>1.59E-05</td><td>uc003wfr.3,uc003wft.3,uc003wfu.2,uc011kup.1</td></tr>
<tr><td>?|26823</td><td>1</td><td>2.10E-07</td><td>uc011mlh.1</td></tr>
<tr><td>?|280660</td><td>0</td><td>0</td><td>uc010nib.1</td></tr>
<tr><td>?|317712</td><td>0</td><td>0</td><td>uc010ihw.1</td></tr>
<tr><td>?|340602</td><td>0</td><td>0</td><td>uc004dpj.2</td></tr>
<tr><td>?|388795</td><td>7</td><td>1.54E-07</td><td>uc010zub.1</td></tr>
<tr><td>?|390284</td><td>13</td><td>5.85E-06</td><td>uc001qoa.2</td></tr>
<tr><td>?|391343</td><td>7</td><td>2.18E-07</td><td>uc010ewg.2,uc010ewh.1</td></tr>
<tr><td>?|391714</td><td>0</td><td>0</td><td>uc011cjl.1</td></tr>
<tr><td>?|404770</td><td>0</td><td>0</td><td>uc010mpu.1</td></tr>
<tr><td>?|441362</td><td>0</td><td>0</td><td>uc003ydl.1,uc010mgi.2</td></tr>
<tr><td>?|442388</td><td>0</td><td>0</td><td>uc011lec.1</td></tr>
<tr><td>?|553137</td><td>1652</td><td>5.98E-05</td><td>uc003prp.2</td></tr>
<tr><td>?|57714</td><td>1785</td><td>3.01E-05</td><td>uc002jye.1,uc002jyf.2,uc002jyg.1</td></tr>
<tr><td>?|645851</td><td>54</td><td>6.27E-06</td><td>uc010crq.1</td></tr>
<tr><td>?|652919</td><td>5.9</td><td>1.56E-07</td><td>uc010yxg.1</td></tr>
<tr><td>?|653553</td><td>290</td><td>0.000102858</td><td>uc011lsc.1</td></tr>
<tr><td>?|728045</td><td>0</td><td>0</td><td>uc011cbi.1</td></tr>
<tr><td>?|728603</td><td>0</td><td>0</td><td>uc001jfy.3</td></tr>
<tr><td>?|728788</td><td>0</td><td>0</td><td>uc010mnx.1</td></tr>
<tr><td>?|729884</td><td>0</td><td>0</td><td>uc003hdz.3</td></tr>
<tr><td>?|8225</td><td>258</td><td>2.09E-05</td><td>uc004cpd.1,uc004cpe.1,uc011mgy.1</td></tr>
<tr><td>?|90288</td><td>77</td><td>3.21E-06</td><td>uc003emg.2</td></tr>
<tr><td>A1BG|1</td><td>206.61</td><td>1.00E-05</td><td>uc002qsd.3,uc002qsf.1</td></tr>
<tr><td>A1CF|29974</td><td>0</td><td>0</td><td>uc001jjh.2,uc001jji.2,uc001jjj.2,uc001jjk.1,uc009xov.2,uc010qhn.1,</td></tr>
</table>

<table>
<tr><th>gene_id</th><th>normalized_count</th></tr>
<tr><td>?|100130426</td><td>0</td></tr>
<tr><td>?|100133144</td><td>20.283</td></tr>
<tr><td>?|100134869</td><td>30.3173</td></tr>
<tr><td>?|10357</td><td>130.5489</td></tr>
<tr><td>?|10431</td><td>816.8954</td></tr>
<tr><td>?|136542</td><td>0</td></tr>
<tr><td>?|155060</td><td>272.7273</td></tr>
<tr><td>?|26823</td><td>0.4288</td></tr>
<tr><td>?|280660</td><td>0</td></tr>
<tr><td>?|317712</td><td>0</td></tr>
<tr><td>?|340602</td><td>10.2916</td></tr>
<tr><td>?|388795</td><td>0</td></tr>
<tr><td>?|390284</td><td>6.0034</td></tr>
<tr><td>?|391343</td><td>16.7238</td></tr>
<tr><td>?|391714</td><td>0.8576</td></tr>
<tr><td>?|404770</td><td>0</td></tr>
<tr><td>?|441362</td><td>0</td></tr>
<tr><td>?|442388</td><td>0</td></tr>
<tr><td>?|553137</td><td>989.7084</td></tr>
<tr><td>?|57714</td><td>409.9485</td></tr>
<tr><td>?|645851</td><td>15.0086</td></tr>
<tr><td>?|652919</td><td>0</td></tr>
<tr><td>?|653553</td><td>66.4666</td></tr>
<tr><td>?|728045</td><td>0</td></tr>
<tr><td>?|728603</td><td>0</td></tr>
<tr><td>?|728788</td><td>4.717</td></tr>
<tr><td>?|729884</td><td>0</td></tr>
<tr><td>?|8225</td><td>592.6244</td></tr>
<tr><td>?|90288</td><td>510.2916</td></tr>
<tr><td>A1BG|1</td><td>97.9374</td></tr>
<tr><td>A1CF|29974</td><td>0</td></tr>
</table>

[4] https://www.biostars.org/p/106127/

BED output format: Tab separated BED file, in which the RNA-seq V2 Gene file is converted, with the following fields:

1. **chrom** (retrieved from NCBI Entrez Gene database[5], according to the entrez gene id provided in field 6, e.g., “chr4”)
2. **chromStart** (retrieved from NCBI Entrez Gene database[5], according to the entrez gene id provided in field 6, e.g., 109740692)
3. **chromEnd** (retrieved from NCBI Entrez Gene database[5], according to the entrez gene id provided in field 6, e.g., 109802225)
4. **strand** (retrieved from NCBI Entrez Gene database[5], according to the entrez gene id provided in field 6, e.g., ‘-’)
5. **gene_symbol** (retrieved from the 1. field of the TCGA RNA-Seq V2 gene results file, part just before “|”; it represents the symbol of the quantified gene, e.g., “CFI”)
6. **entrez_gene_id** (retrieved from the 1. field of the TCGA RNA-Seq V2 gene results file, part just after “|”; it represents the Entrez gene ID of the quantified gene, e.g., “3426”)
7. **raw_count**(*) (retrieved from the 2. field of the TCGA RNA-Seq V2 gene results file, e.g., 131.00)
8. **scaled_estimate**(*) (retrieved from the 3. field of the TCGA RNA-Seq V2 gene results file, e.g., 2.18150649130347e-06)
9. **transcript_id** (retrieved from the 4. field of the TCGA RNA-Seq V2 gene results file, this field may include multiple transcript ids delimitated by comma “,”, e.g., uc003hzq.2,uc003hzr.3,uc003hzs.3,uc011cft.1)
10. **normalized_count** (retrieved from the 2. field of the TCGA RNA-Seq V2 gene result normalized file, e.g., 45.1503)

[5] All the NCBI queries are performed according to the following rest query and by taking into account the GRCh37 (hg19) reference genome: http://eutils.ncbi.nlm.nih.gov/entrez/eutils/efetch.fcgi/?db=Gene&id=ID

Rsem isoform results & Rsem isoform normalized results

Input:
One tab delimited file is provided by TCGA for each aliquot, with the following fields:

1. Isoform_id (i.e., the UCSC database ID of the isoform whose expression is quantified)
2. Raw_count(*) (i.e., see "Important remarks" on page 20)
3. Scaled_estimate(*) (i.e., see "Important remarks" on page 20)

Each row in the input file refers to a single isoform.

Input normalized:
Another tab delimited file is provided by TCGA for each aliquot, with the following fields:

1. Isoform_id (i.e., the UCSC database ID of the isoform whose expression is quantified)
2. Normalized_count (i.e., RSEM expression estimates normalized to set the upper quartile count at 300 for isoform level estimates, by dividing all "raw_count" values by the 75th percentile of the raw counts (after removing zeros) and multiply that by 1000[6])

Each row in the input file refers to a single isoform.

We merge the two original TCGA files in one single BED file by adding the normalized_count field after the other fields.

isoform_id	raw_count	scaled_estimate
uc011lsn.1	0	0
uc010unu.1	47.3	2.15E-06
uc010uoa.1	0	0
uc002bgz.2	70.7	2.30E-06
uc002bic.2	0	0
uc010zzl.1	304.44	3.39E-05
uc001jiu.2	1905	9.07E-05
uc010qhg.1	0	0
uc011krn.1	0	0
uc003wfr.3	199.19	3.34E-06
uc003wft.3	103.74	1.71E-06
uc003wfu.2	321.5	4.95E-06
uc011kup.1	11.57	6.95E-07
uc011mlh.1	1	1.43E-07
uc010nib.1	0	0
uc010ihw.1	0	0
uc004dpj.2	24	6.30E-07
uc010zub.1	0	0
uc001qoa.2	14	5.76E-06
uc010ewg.2	7.44	1.80E-07
uc010ewh.1	31.56	4.73E-07
uc011cjl.1	2	7.57E-08
uc010mpu.1	0	0
uc003ydl.1	0	0
uc010mgi.2	0	0
uc011lec.1	0	0
uc003prp.2	2308	4.60E-05
uc002jye.1	13.13	6.73E-07
uc002jyf.2	457.31	3.98E-06
uc002jyg.1	485.56	4.72E-06
uc010crq.1	35	2.46E-06
uc010yxg.1	0	0

isoform_id	normalized_count
uc011lsn.1	0
uc010unu.1	22.544
uc010uoa.1	0
uc002bgz.2	33.6969
uc002bic.2	0
uc010zzl.1	145.1016
uc001jiu.2	907.9571
uc010qhg.1	0
uc011krn.1	0
uc003wfr.3	94.9375
uc003wft.3	49.4443
uc003wfu.2	153.2327
uc011kup.1	5.5145
uc011mlh.1	0.4766
uc010nib.1	0
uc010ihw.1	0
uc004dpj.2	11.4388
uc010zub.1	0
uc001qoa.2	6.6727
uc010ewg.2	3.546
uc010ewh.1	15.0421
uc011cjl.1	0.9532
uc010mpu.1	0
uc003ydl.1	0
uc010mgi.2	0
uc011lec.1	0
uc003prp.2	1100.0342
uc002jye.1	6.258
uc002jyf.2	217.9621
uc002jyg.1	231.4266
uc010crq.1	16.6816
uc010yxg.1	0

[6] https://www.biostars.org/p/106127/

BED output format: Tab separated BED file, in which the RNA-seq V2 isoform file is converted, with the following fields:

1. **chrom** (retrieved from NCBI Entrez Gene database[7], according to the entrez gene id retrieved from UCSC database[8] based on the UCSC *isoforms_id* provided in 1. field of the TCGA RNA-Seq isoform results V2 file, e.g., “chr17”)
2. **chromStart** (retrieved from NCBI Entrez Gene database[7], according to the entrez gene id retrieved from UCSC database[8] based on the UCSC *isoforms_id* provided in 1. field of the TCGA RNA-Seq isoform results V2 file , e.g., 8310238)
3. **chromEnd** (retrieved from NCBI Entrez Gene database[7], according to the entrez gene id retrieved from UCSC database[8] based on the UCSC *isoforms_id* provided in 1. field of the TCGA RNA-Seq isoform results V2 file, e.g., 8322516)
4. **strand** (retrieved from NCBI Entrez Gene database[7], according to the entrez gene id retrieved from UCSC database[8] based on the UCSC *isoforms_id* provided in 1. field of the TCGA RNA-Seq isoform results V2 file e.g., ‘+’)
5. **gene_symbol** (retrieved from NCBI Entrez Gene database[7], according to the entrez gene id retrieved from UCSC database[8] based on the UCSC *isoforms_id* provided in 1. field of the TCGA RNA-Seq isoform results V2 file, e.g., “ARHGEF15”)
6. **entrez_gene_id** (retrieved from NCBI Entrez Gene database[7], according to the entrez gene id retrieved from UCSC database[8] based on the UCSC *isoforms_id* provided in 1. field of the TCGA RNA-Seq isoform results V2 file, e.g., “22899”)
7. **transcript_id** (retrieved from the 1. field “*isoform_id”* of the TCGA RNA-Seq V2 isoform results file, e.g., uc002glb.1)
8. **raw_count**(*) (retrieved from the 2. field of the TCGA RNA-Seq V2 isoform results file, e.g., 8.31)
9. **scaled_estimate**(*) (retrieved from the 3. field of the TCGA RNA-Seq isoform results V2 file, e.g., 1.42848669335672e-07)
10. **normalized_count** (retrieved from the 2. field of the TCGA RNA-Seq V2 isoform result normalized file, e.g., 3.2186)

(*) Important remarks:

[http://seqanswers.com/forums/showthread.php?t=42911]

[https://wiki.nci.nih.gov/display/TCGA/RNASeq+Version+2]

Raw count represents the (estimated) number of reads that aligned to a transcript (in the case of Rsem genes or Rsem isoforms). This value is not an integer because RSEM only reports a guess of how many ambiguously mapping reads belong to a transcript/gene. This number is what the TCGA slightly misleadingly calls raw counts.

The **scaled estimate** value is the estimated frequency of the gene/transcript amongst the total number of transcripts that were sequenced. Newer versions of RSEM call this value (multiplied

[7] All the NCBI queries are performed according to the following rest query and by taking into account the GRCh37 (hg19) reference genome: http://eutils.ncbi.nlm.nih.gov/entrez/eutils/efetch.fcgi/?db=Gene&id=ID
[8] Used data from the University of California at Santa Clara (UCSC) Genome Browser database are retrieved from http://hgdownload.cse.ucsc.edu/goldenPath/hg19/database/knownGene.txt.gz

by 1e6) TPM - Transcripts Per Million. It’s closely related to FPKM, as explained on the RSEM website (http://deweylab.biostat.wisc.edu/rsem/rsem-calculate-expression.html#output). The important point is that TPM, like FPKM, is independent of transcript length, whereas "raw" counts are not.

The *.normalized_results files just contain a scaled version of the raw_count column. The values are divided by the 75-percentile and multiplied by 1000. This should make the values a bit more comparable between experiments.

The files use Universally Unique Identifier (UUID) as patient id and as file name, which is a randomly-generated, 32-digit hexadecimal value. There is a repository for converting a UUID to a conventional TCGA patient’s id (TCGA barcode) available at the RNA-Seq V2 meta data files.

miRNA-Seq

miRNA-Seq data are derived from the sequencing of micro RNAs (miRNA); they contain information about both nucleotide sequence and expression.
More details are available at https://wiki.nci.nih.gov/display/TCGA/miRNASeq

Two files are provided by TCGA for each aliquot:
- miRNA quantification (i.e., the calculated expression for all reads aligning to a particular miRNA)
- Isoform quantification (i.e., the calculated expression for each individual miRNA sequence isoform observed)

miRNA quantification

Input:
One tab delimited file is provided by TCGA for each aliquot, with the following fields:
1. miRNA_ID (i.e., a valid miRBase ID (http://www.mirbase.org/))
2. read_count (i.e., the sum of fractions of reads that mapped to a miRNA)
3. reads_per_million_miRNA_mapped (i.e., millions of reads that mapped to a miRNA)
4. cross-mapped (i.e., cross-mapped to other miRNA forms (Y or N))

Each row in the input file refers to a single miRNA.

miRNA_ID	read_count	reads_per_million_miRNA_mapped	cross-mapped
hsa-let-7a-1	76213	13484.031491	N
hsa-let-7a-2	151321	26772.560183	Y
hsa-let-7a-3	77498	13711.380899	N
hsa-let-7b	85979	15211.886995	N
hsa-let-7c	11107	1965.112747	Y
hsa-let-7d	9740	1723.255438	N
hsa-let-7e	15161	2682.369168	N
hsa-let-7f-1	261	46.177584	N
hsa-let-7f-2	94960	16800.855895	N
hsa-let-7g	6601	1167.885950	N
hsa-let-7i	1550	274.234695	N
hsa-mir-1-1	0	0.000000	N
hsa-mir-1-2	30	5.307768	N
hsa-mir-100	1677	296.704247	N
hsa-mir-101-1	45395	8031.538051	N
hsa-mir-101-2	377	66.700955	N
hsa-mir-103-1	126526	22385.689691	Y
hsa-mir-103-2	57	10.084760	N
hsa-mir-105-1	1	0.176926	N
hsa-mir-105-2	2	0.353851	N
hsa-mir-106a	11	1.946182	Y
hsa-mir-106b	1060	187.541146	N
hsa-mir-107	143	25.300362	Y
hsa-mir-10a	195986	34674.942539	N
hsa-mir-10b	1655780	292949.885998	N
hsa-mir-1178	0	0.000000	N
hsa-mir-1179	2	0.353851	N
hsa-mir-1180	258	45.646807	N

BED output format: Tab separated BED file, in which the miRNA-seq Mirna quantification file is converted, with the following fields:

1. **chrom** (retrieved from miRBase database[9], according to the miRNA id provided in field 5, e.g., “chr9”)
2. **chromStart** (retrieved from miRBase database[9], according to the miRNA id provided in field 5, e.g., 94175957)
3. **chromEnd** (retrieved from miRBase database[9], according to the miRNA id provided in field 5, e.g., 94176036)
4. **strand** (retrieved from miRBase database[9], according to the miRNA id provided in field 5, e.g., ‘+’)
5. **mirna_id** (retrieved from the 1. field of the TCGA miRNA-Seq file, e.g., “hsa-let-7a-1”)
6. **read_count** (retrieved from the 2. field of the TCGA miRNA -Seq file, e.g., 29726)
7. **reads_per_million_miRNA_mapped** (retrieved from the 3. field of the TCGA miRNA -Seq file, e.g., 12429.699816)
8. **cross-mapped** (retrieved from the 4. field of the TCGA miRNA -Seq file, e.g., ‘N’)

Isoform quantification

Input:

One tab delimited file is provided by TCGA for each aliquot, with the following fields:

1. miRNA_ID (i.e., a valid miRBase ID (http://www.mirbase.org/))
2. isoform_coords (i.e., Alignment coordinates as <version>:<Chromosome>:<Start position>-<End position>:<Strand>)
3. read_count (i.e., raw read count)
4. reads_per_million_miRNA_mapped (i.e., millions of reads that mapped to a miRNA)
5. cross-mapped (i.e., cross-mapped to other miRNA forms (Y or N))
6. miRNA_region (i.e., miRBase accession number[9] of a class of miRNA sequence, e.g., mature, stemloop, ...)

Each row in the input file refers to a single isoform.

[9] Used hg19 data are retrieved from the version 20 of the miRBase database at ftp://mirbase.org/pub/mirbase/20/

miRNA_ID	isoform_coords	read_count	reads_per_million_miRNA_mapped	cross-mapped	miRNA_region
hsa-let-7a-1	hg19:9:96938243-96938264:+	4	0.707702	N	mature,MIMAT0000062
hsa-let-7a-1	hg19:9:96938243-96938265:+	14	2.476959	N	mature,MIMAT0000062
hsa-let-7a-1	hg19:9:96938243-96938266:+	82	14.507900	N	mature,MIMAT0000062
hsa-let-7a-1	hg19:9:96938243-96938267:+	5	0.884628	N	mature,MIMAT0000062
hsa-let-7a-1	hg19:9:96938244-96938263:+	114	20.169520	N	mature,MIMAT0000062
hsa-let-7a-1	hg19:9:96938244-96938264:+	4633	819.696350	N	mature,MIMAT0000062
hsa-let-7a-1	hg19:9:96938244-96938265:+	10671	1887.973181	N	mature,MIMAT0000062
hsa-let-7a-1	hg19:9:96938244-96938266:+	58860	10413.841386	N	mature,MIMAT0000062
hsa-let-7a-1	hg19:9:96938244-96938267:+	1567	277.242430	N	mature,MIMAT0000062
hsa-let-7a-1	hg19:9:96938244-96938268:+	61	10.792462	N	mature,MIMAT0000062
hsa-let-7a-1	hg19:9:96938245-96938264:+	3	0.530777	N	mature,MIMAT0000062
hsa-let-7a-1	hg19:9:96938245-96938265:+	1	0.176926	N	mature,MIMAT0000062
hsa-let-7a-1	hg19:9:96938245-96938266:+	8	1.415405	N	mature,MIMAT0000062
hsa-let-7a-1	hg19:9:96938247-96938265:+	1	0.176926	N	mature,MIMAT0000062
hsa-let-7a-1	hg19:9:96938247-96938266:+	12	2.123107	N	mature,MIMAT0000062
hsa-let-7a-1	hg19:9:96938266-96938290:+	1	0.176926	N	stemloop
hsa-let-7a-1	hg19:9:96938295-96938315:+	16	2.830810	N	star,MIMAT0004481
hsa-let-7a-1	hg19:9:96938295-96938316:+	53	9.377057	N	star,MIMAT0004481
hsa-let-7a-1	hg19:9:96938295-96938317:+	76	13.446346	N	star,MIMAT0004481
hsa-let-7a-1	hg19:9:96938295-96938318:+	5	0.884628	N	star,MIMAT0004481
hsa-let-7a-1	hg19:9:96938296-96938317:+	2	0.353851	N	star,MIMAT0004481
hsa-let-7a-1	hg19:9:96938296-96938318:+	22	3.892363	N	star,MIMAT0004481
hsa-let-7a-1	hg19:9:96938297-96938318:+	2	0.353851	N	star,MIMAT0004481
hsa-let-7a-2	hg19:11:122017231-122017253:-	8	1.415405	N	star,MIMAT0010195
hsa-let-7a-2	hg19:11:122017274-122017298:-	24	4.246215	N	mature,MIMAT0000062

BED output format: Tab separated BED file, in which the miRNA-seq Isoform quantification file is converted, with the following fields:

1. **chrom** (retrieved from the 2. field of the TCGA miRNA-Seq file, part just after the first ":", e.g., "9")
2. **chromStart** (retrieved from the 2. field of the TCGA miRNA-Seq file, part just after the second ":", e.g.,96938243)
3. **chromEnd** (retrieved from the 2. field of the TCGA miRNA-Seq file, part just before the third ":", e.g., 96938264)
4. **strand** (retrieved from the 2. field of the TCGA miRNA-Seq file, part just after the third ":", e.g., '+')
5. **genome_version** (retrieved from the 2. field of the TCGA miRNA-Seq file, part just before the first ":", e.g., "hg19")
6. **mirna_id** (retrieved from the 1. field of the TCGA miRNA-Seq file, e.g., "has-let-7a-1")
7. **read_count** (retrieved from the 3. field of the TCGA miRNA-Seq file, e.g., 4)
8. **reads_per_million_miRNA_mapped** (retrieved from the 4. field of the TCGA miRNA-Seq file, e.g., 0.707702)
9. **cross-mapped** (retrieved from the 5. field of the TCGA miRNA-Seq file, e.g., 'N')
10. **miRNA_region** (retrieved from the 6. field of the TCGA miRNA-Seq file, e.g., "mature, MIMAT0000062")

Copy Number Variation

A copy number variation (CNV) is the number of copies of a given gene per cell.
More details are available at https://wiki.nci.nih.gov/display/TCGA/SNP+array-based+data.

Two files are provided by TCGA for each aliquot:
- hg19.seg (includes both germline and somatic CNVs)
- nocnv_hg19.seg (includes only somatic CNVs)

Input:
Two tab delimited files are provided by TCGA for each aliquot, with the following fields:
1. Sample (i.e., the TCGA internal sample ID)
2. Chromosome (i.e., the name or number of the chromosome where the CNV is located)
3. Start (i.e., the starting position of the CNV feature in the chromosome)
4. End (i.e., the ending position of the CNV feature in the chromosome)
5. Num_Probes (i.e., the number of consecutive probes that comprise the genome segment with the CNV)
6. Segment_Mean (i.e., the estimated Copy Number (CN) ratio for the segment, that is the $\log_2$ ratio of the tumor intensity of CN to the normal intensity of CN; use $(2^{Segment\_Mean}) * 2$ to convert to absolute CN)[10]

Each row in the input file refers to a single CNV.

Sample	Chromosome	Start	End	Num_Probes	Segment_Mean
AQUAE_p_TCGA_112_304_b2_N_GenomeWideSNP_6_A01_1348356	1	61735	1628826	229	0.1756
AQUAE_p_TCGA_112_304_b2_N_GenomeWideSNP_6_A01_1348356	1	1642103	1688058	20	0.8677
AQUAE_p_TCGA_112_304_b2_N_GenomeWideSNP_6_A01_1348356	1	1688192	16149915	8139	0.0169
AQUAE_p_TCGA_112_304_b2_N_GenomeWideSNP_6_A01_1348356	1	16153497	16154239	8	1.105
AQUAE_p_TCGA_112_304_b2_N_GenomeWideSNP_6_A01_1348356	1	16154966	25570830	5697	0.0116
AQUAE_p_TCGA_112_304_b2_N_GenomeWideSNP_6_A01_1348356	1	25571269	25696602	56	-0.4542
AQUAE_p_TCGA_112_304_b2_N_GenomeWideSNP_6_A01_1348356	1	25698469	35091674	4921	0.0113
AQUAE_p_TCGA_112_304_b2_N_GenomeWideSNP_6_A01_1348356	1	35102654	35104491	20	-0.608
AQUAE_p_TCGA_112_304_b2_N_GenomeWideSNP_6_A01_1348356	1	35114268	72768916	23688	0.0027
AQUAE_p_TCGA_112_304_b2_N_GenomeWideSNP_6_A01_1348356	1	72768936	72811133	44	-1.8052
AQUAE_p_TCGA_112_304_b2_N_GenomeWideSNP_6_A01_1348356	1	72811148	76050844	1908	-0.0045
AQUAE_p_TCGA_112_304_b2_N_GenomeWideSNP_6_A01_1348356	1	76054763	76054854	2	-2.6875
AQUAE_p_TCGA_112_304_b2_N_GenomeWideSNP_6_A01_1348356	1	76059509	86573546	7067	-0.0077
AQUAE_p_TCGA_112_304_b2_N_GenomeWideSNP_6_A01_1348356	1	86573802	86577211	2	-2.1489
AQUAE_p_TCGA_112_304_b2_N_GenomeWideSNP_6_A01_1348356	1	86577870	99732202	8251	0.0046
AQUAE_p_TCGA_112_304_b2_N_GenomeWideSNP_6_A01_1348356	1	99732737	99737222	2	-1.956
AQUAE_p_TCGA_112_304_b2_N_GenomeWideSNP_6_A01_1348356	1	99737524	104163499	2699	0.003
AQUAE_p_TCGA_112_304_b2_N_GenomeWideSNP_6_A01_1348356	1	104163787	104303403	27	-0.7798
AQUAE_p_TCGA_112_304_b2_N_GenomeWideSNP_6_A01_1348356	1	104303501	110224427	3562	-0.0077
AQUAE_p_TCGA_112_304_b2_N_GenomeWideSNP_6_A01_1348356	1	110225642	110232974	14	-0.5318
AQUAE_p_TCGA_112_304_b2_N_GenomeWideSNP_6_A01_1348356	1	110233053	110240178	14	-1.2134
AQUAE_p_TCGA_112_304_b2_N_GenomeWideSNP_6_A01_1348356	1	110242953	152759678	10146	0.009
AQUAE_p_TCGA_112_304_b2_N_GenomeWideSNP_6_A01_1348356	1	152761923	152768700	37	-1.5703
AQUAE_p_TCGA_112_304_b2_N_GenomeWideSNP_6_A01_1348356	1	152773905	161479438	5226	0.0031
AQUAE_p_TCGA_112_304_b2_N_GenomeWideSNP_6_A01_1348356	1	161496900	161648237	56	0.847
AQUAE_p_TCGA_112_304_b2_N_GenomeWideSNP_6_A01_1348356	1	161648621	210071062	32856	0.0011
AQUAE_p_TCGA_112_304_b2_N_GenomeWideSNP_6_A01_1348356	1	210081613	210083984	3	-2.6172
AQUAE_p_TCGA_112_304_b2_N_GenomeWideSNP_6_A01_1348356	1	210086552	222366668	8539	-1e-04

[10] https://www.biostars.org/p/112310/

BED output format: Tab separated BED file, in which the CNV_file is converted, with the following fields:

1. **chrom** (retrieved from the 2. field of the TCGA CNV file, e.g., “1”)
2. **chromStart** (retrieved from the 3. field of the TCGA CNV file, e.g., 61735)
3. **chromEnd** (retrieved from the 4. field of the TCGA CNV file, e.g., 1628826)
4. **strand** (unknown, set to ‘*’)
5. **Num_Probes** (retrieved from the 5. field of the TCGA CNV file, e.g., 229)
6. **Segment_Mean** (retrieved from the 6. field of the TCGA CNV file, e.g., 0.1756)
7. **is_nocnv** (’Y’ if the current line refers to the “nocnv_hg19.seg” file, ‘N’ otherwise)

Clinical and specimen (meta) data

Meta data are data about data. Within TCGA, meta data are referred to as clinical data or specimen data. Clinical data contain several attributes related to the clinical history of the patient and of the patient's sample. Specimen data contain attributes that concern the collection of the sample and the experimental procedure. TCGA meta data attributes are subdivided in groups which specify the type of attribute (e.g., clinical_drug, clinical_follow_up, clinical_patient, clinical_radiation, … or biospecimen_aliquot, biospecimen_analyte, biospecimen_diagnostic_slides, …).

Input:
One or more tab delimited files are provided by TCGA for all patients of a particular tumor (e.g., breast cancer), which contain several clinical, demographic, specimen attributes.

bcr_patient_barcode	bcr_patient_uuid	gender	menopause_status
TCGA-AR-A1AR	eda6d2d5-4199-4f76-a45b-1d0401b4e54c	FEMALE	Post
TCGA-BH-A1EO	4510295e-8aa7-4ef1-b2b7-91cc902f8200	FEMALE	Post
TCGA-BH-A1ES	51ccbf1b-7cae-44ba-991a-11eda8b8c404	FEMALE	Pre
TCGA-BH-A1ET	8986a141-eae7-4157-b695-02cc6fc3b071	FEMALE	Pre
TCGA-BH-A1EU	a1093598-d3a8-4ffe-83fc-bc7d1faff7e5	FEMALE	Post
TCGA-BH-A1EV	417dea5f-f68e-4dab-940e-43ae8c67e5e6	FEMALE	Pre
TCGA-BH-A1EW	9d166970-07c8-4ca3-9cfa-ed0049df9ecc	FEMALE	Pre
TCGA-BH-A1F0	21ef1730-e5a7-47ce-b419-d000bb59ae15	FEMALE	Post
TCGA-C8-A1HF	a2453bcb-90f2-4505-949d-a89cf4bfc9b8	Available	Post
TCGA-C8-A1HG	0c23c380-363c-474d-b64c-b47f612a8225	Available	Post
TCGA-C8-A1HI	444374f8-9282-439c-af00-0f828edcbff3	Available	Pre
TCGA-C8-A1HL	a8a199c9-d781-4e6c-af8c-c85d60c7cd40	Available	Pre

Output:
One meta data attribute tab delimited file for each patient (.meta), whose rows contain all the meta data attribute-value pairs for the specific patient, with each attribute fully specified through the pipe (|) delimited composition of the name of the group it belongs to and the name of the attribute (i.e., group_name|attribute_name, e.g., clinical_patient|tumor_tissue_site).
For example the meta data file for the patient TCGA-AR-A1AR (TCGA-AR-A1AR.meta) contains:

```
biospecimen_aliquot|bcr_patient_uuid      eda6d2d5-4199-4f76-a45b-1d0401b4e54c
clinical_patient|gender                   female
clinical_patient|tumor_status             tumor free
…
```

We added also some additional meta data attributes, within a specific group named `manually_curated`, and their values retrieved from the directory structure, filenames, etc. of TCGA; the main ones are the following:

- `id tcga-07-0227-20a-01d-a39x-05`
- `dataType RNASeq`
- `exp_data_bed_url ftp://bioinf.iasi.cnr.it/bed/prad/cnv/TCGA-2A-A8VL-01A-21D-A376-01.bed`
- `exp_metadata_url ftp://bioinf.iasi.cnr.it/bed/prad/cnv/TCGA-2A-A8VL-01A-21D-A376-01.bed.meta`
- `md5sum ad3fdd0de6887559604a95014984a12d`
- `rna_seq_data_unit RPKM | RSEM | ...`
- `rna_seq_exp_type exon_quantification | gene_quantification | splice_junction_quantification`
- `seqPlatform Illumina_GA2 | Illumina_GA2e | Illumina_GA2x | Illumina_HiSeq_2000`
- `tissue_status tumoral | control | normal`
- `tumor_description Bladder Urothelial Carcinoma | ...`
- `tumor_tag BLCA | ...`

Additional output files

We also provide the following output files:

Meta data dictionary file

One meta data dictionary tab delimited file, which contains all the possible values of any meta data attribute, for example:

menopause_status
    Pre (<6 months since LMP AND no prior bilateral ovariectomy AND not on estrogen replacement)
    Peri (6-12 months since last menstrual period)
    [Unknown]
    Post (prior bilateral ovariectomy OR >12 mo since LMP with no prior hysterectomy)
    CDE_ID:2957270
histologic_diagnosis_other
    Mixed infiltrating lobular and grade 1 ductal carcinoma
    MUCINOUS & PAPILLARY
    CDE_ID:3124492
    Lobular carcinoma with ductal features
    ductal/lobular
    IDC+ mucinous carcinoma
    Ductal/Lobular
    Infiltrating ductal & lobular
    Infiltrating ductal and lobular carcinoma
    ductal and lobular
    Invasive ductal and lobular carcinoma
    lobular/ductal
    Mixed invasive ductal and invasive lobular
    Lobular/Ductal
    [Not Applicable]
    Mixed diagnosis
    with ductal and lobular phenotypes
    invasive ductal and lobular carcinoma

When performing batch conversions, a meta data dictionary file is generated for

1. all the converted data;
2. each experiment, i.e., DNA-Seq, DNA methylation, RNA-seq (V1 and V2), miRNA-seq, and CNV;
3. each tumor.

Meta data information files

We output a comma separated values (CSV) file containing the occurrences of all the meta data attributes related to each tumor type ("*meta2disease_table.csv*").

Additionally, we generate the following additional output files for each tumor:

- a CSV file containing the occurrence counts of each meta data attribute related to the tumor ("*meta2dataType_table.csv*")
- a CSV file containing a table with a list of all meta data attributes concatenated with all possible values on the rows and the list of all available data types for the considered tumor; a generic cell of this table contains the occurrences of the pair attribute|value in a specific data type ("*meta_values2dataTypes_table.csv*")
- a tab separated values (TSV) file containing a list of all meta data attributes concatenated with all possible values followed by the occurrences of this pair (attribute|value) in all data types for the considered tumor ("*meta_values2sample_list.tsv*")

Experiment information files

We generate an additional output file for each subtype of all the DNA-seq, DNA methylation, RNA-seq (V1 and V2), miRNA-seq, and CNV data types, called "*exp_info.tsv*". It is a tab delimited files that includes:

- number of aliquots;
- number of samples (tissue);
- number of patients.

Annotations files

We provide an additional output file for each subtype of RNA-seq (V1 and V2) experiments, called "*annotations.tsv*". It is a tab delimited file that contains the following fields for each gene in the considered genomic experiment:

- gene symbol;
- entrez gene id;
- lists of gene transcript ids (only for the gene data subtype).

It is worth noting that each subtype has the same annotations.

Additional data file formats

Besides the BED format, to ensure maximum usage, we also support the set of additional data file formats following specified.

CSV format

The standard Comma Separated Values (CSV) file format defines the structure and content of the experimental data files as equal to the ones of the BED format, but a comma (instead of a tabulator) is used to separate the different fields.
The structure of the meta data files is the same as for the BED format.

XML format

The standard eXtended Markup Language (XML) file format defines the content of the experimental data files as equal to the one of the BED format, but the file structure is designed according to the XML style. In particular, we define one experimental XML file for each aliquot; the content of this file starts with the XML heading line

```
<?xml version="1.0" encoding="UTF-8"?>
```

and with the root node called `<aliquot>`.
Then, for each experimental measure (row of the input TCGA data file) we define a `<data>` tag containing the experimental fields as sub-tags and their values.
In the following, we provide an example of XML file of DNA methylation:

```
<?xml version="1.0" encoding="UTF-8"?>
<aliquot>
      <data>
            <chr>chr17</chr>
            <start>62503072</start>
            <stop>62503072</stop>
            <strand>+</strand>
            <composite_element_ref>cg00003784</composite_element_ref>
            <beta_value>0.0286291327274318</beta_value>
            <gene_symbol>CEP95</gene_symbol>
      </data>
      <data>
            <chr>chr19</chr>
            <start>17336525</start>
            <stop>17336525</stop>
            <strand>+</strand>
            <composite_element_ref>cg00003818</composite_element_ref>
            <beta_value>null</beta_value>
            <gene_symbol>OCEL1</gene_symbol>
```

```
      </data>
      …
</aliquot>
```

The structure of the meta data files is the same as for the BED format.

JSON format

The standard JavaScript Object Notation (JSON) format defines the content of the experimental data files as equal to the one of the BED format, but the file structure is designed according to the JSON style. In particular, we define one experimental JSON file for each aliquot; the content of this file starts with the root node called `"aliquot"`.

Then, for each experimental measure (row of the input TCGA data file) we define a `"data"` tag containing the experimental fields as sub-tags and their values.

In the following, we provide an example of JSON file of DNA methylation:

```
{
      "aliquot": {
            "data": [
                  {
                        "chr": "chr17",
                        "start": "62503072",
                        "stop": "62503072",
                        "strand": "+",
                        "composite_element_ref": "cg00003784",
                        "beta_value": "0.0286291327274318",
                        "gene_symbol": "CEP95"
                  },
                  {
                        "chr": "chr19",
                        "start": "17336525",
                        "stop": "17336525",
                        "strand": "+",
                        "composite_element_ref": "cg00003818",
                        "beta_value": "null",
                        "gene_symbol": "OCEL1"
                  },
….
}
```

The structure of the meta data files is the same as for the BED format.

GTF format

The bioinformatics standard Gene Transfer Format (GTF) defines the content of the experimental data files as equal to the one of the BED format, but the file structure is designed according to the GTF style. In particular, we define one experimental GTF file for each aliquot.

The nine tab-separated GTF fields are[11]:

1. **seqname** - the name of the sequence; it must be a chromosome or scaffold (in our case, the chromosome).
2. **source** - the program that generated this feature (in our case, TCGA2BED)
3. **feature** - the name of this type of feature; some examples of standard feature types are "CDS", "start_codon", "stop_codon", and "exon" (in our case, "TCGA_Region").
4. **start** - the starting position of the feature in the sequence; the first base is numbered 1.
5. **end** - the ending position of the feature in the sequence (inclusive).
6. **score** - a score between 0 and 1000. In UCSC Genome Browser, if the track line *useScore* attribute is set to 1 for this annotation data set, the *score* value determines the level of gray in which this feature is displayed (higher numbers = darker gray). If there is no score value, "." is entered.
7. **strand** - valid entries include '+', '-', or '.' (for don't know/don't care).
8. **frame** - if the feature is a coding exon, *frame* should be a number between 0 and 2 that represents the reading frame of the first base; if the feature is not a coding exon, the value should be '.'.
9. **group** - a list of attributes; each attribute consists of a type/value pair (in our case, we include the fields of the experimental data file and their values, e.g., composite_element_ref "cg00003784"; beta_value "0.0286291327274318"; gene_symbol "CEP95"). Attributes must end in a semi-colon and be separated from any following attribute by exactly one space.

In the following, we provide an example of GTF file of DNA methylation:

```
chr17 TCGA2BED  TCGA_Region 62503072  62503072  . + . composite_element_ref "cg00003784"; beta_value "0.0286291327274318"; gene_symbol "CEP95";
chr19 TCGA2BED  TCGA_Region 17336525  17336525  . + . composite_element_ref "cg00003818"; beta_value "null"; gene_symbol "OCEL1";
chr1  TCGA2BED  TCGA_Region 45080600  45080600  . + . composite_element_ref "cg00003858"; beta_value "null"; gene_symbol "RNF220";
chr3  TCGA2BED  TCGA_Region 108476878 108476878 . - . composite_element_ref "cg00003965"; beta_value "null"; gene_symbol "RETNLB";
chr7  TCGA2BED  TCGA_Region 15725862  15725862  . - . composite_element_ref "cg00003994"; beta_value "0.0493941711402823"; gene_symbol "MEOX2";
chr16 TCGA2BED  TCGA_Region 66586745  66586745  . + . composite_element_ref "cg00004055"; beta_value "0.073911219948775"; gene_symbol "CKLF";
chr3  TCGA2BED  TCGA_Region 36981714  36981714  . - . composite_element_ref "cg00004067"; beta_value "0.965022265629378"; gene_symbol "TRANK1";
chr19 TCGA2BED  TCGA_Region 39898015  39898015  . + . composite_element_ref "cg00004072"; beta_value "0.0999956612897953"; gene_symbol "ZFP36";
chr15 TCGA2BED  TCGA_Region 23034447  23034447  . - . composite_element_ref "cg00000622"; beta_value "0.0143491154061897"; gene_symbol "NIPA2";
chr2  TCGA2BED  TCGA_Region 237027592 237027592 . + . composite_element_ref "cg00004073"; beta_value "null"; gene_symbol "AGAP1";
chr9  TCGA2BED  TCGA_Region 139997924 139997924 . + . composite_element_ref "cg00000658"; beta_value "0.837545212449724"; gene_symbol "MAN1B1";
chr19 TCGA2BED  TCGA_Region 54695678  54695678  . + . composite_element_ref "cg00000714"; beta_value "0.164030705433507"; gene_symbol "TSEN34";
chr6  TCGA2BED  TCGA_Region 25282779  25282779  . + . composite_element_ref "cg00000721"; beta_value "0.956370606771304"; gene_symbol "LRRC16A";
chr3  TCGA2BED  TCGA_Region 128902377 128902377 . - . composite_element_ref "cg00000734"; beta_value "0.0626386186322679"; gene_symbol "CNBP";
chr12 TCGA2BED  TCGA_Region 124086477 124086477 . + . composite_element_ref "cg00000769"; beta_value "0.0233990802366794"; gene_symbol "DDX55";
```

The structure of the meta data files is the same as for the BED format.

[11] https://genome.ucsc.edu/FAQ/FAQformat#format4
