# Supplementary material for: TCGA2BED: extracting, extending, integrating, and querying The Cancer Genome Atlas
Source: BMC Bioinformatics. 2017 Jan 3;18:6. doi: 10.1186/s12859-016-1419-5 (PMC5210259; doi:10.1186/s12859-016-1419-5)
Supplement: Additional file 4 — A pdf file containing the user guide of the TCGA2BED software. (PDF 1085 kb) [file 12859_2016_1419_MOESM4_ESM.pdf]

TCGA2BED

User Guide

Fabio Cumbo, Giulia Fiscon, Stefano Ceri,
Marco Masseroli, Emanuel Weitschek

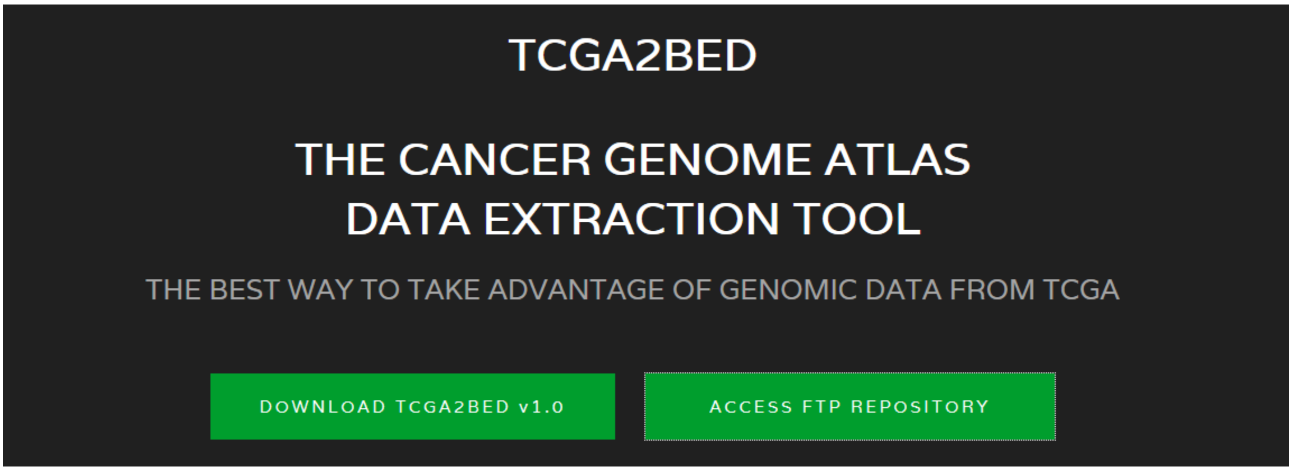

Contents

Introduction 2
TCGA2BED procedure steps 2
Installation 2
JAVA 2
TCGA2BED 2
Executing TCGA2BED 2
Start screen 3
Meta data download 4
Experimental data download 5
Conversion into the supported formats 6
Batch download and conversion into the desired format 10
Data repository 12
Citation 12
Contacts 12
Appendix: tumor tags and tumor names 13

Introduction

This user guide is intended for all the users that want to learn how to use the TCGA2BED tool for downloading and converting TCGA data into the BED, CSV, GTF, JSON, and XML formats. Please refer also to the `TCGA2BED_readme.txt` file (that is included in the software package) for additional details.

TCGA2BED procedure steps

The following steps are necessary to perform the download of public TCGA data and their conversion into the BED, CSV, GTF, JSON, and XML formats. These steps are thoroughly explained in the following sections of this tutorial:

- Meta data download;
- Experimental data download;
- Conversion into the BED, CSV, GTF, JSON, and XML formats.

Installation

JAVA

The TCGA2BED tool requires a working JAVA Virtual Machine (VM) installed. Thus, if not done yet, first download and install the free Oracle JAVA Runtime Environment from http://www.java.com/getjava/.
Several versions for the most common operating systems are available (e.g., Windows x86 for Windows 32 bit, Windows x64 for Windows 64 bit, MacOsX, or Linux). Please choose the right version according to your operating system.

TCGA2BED

You can download and unzip the multi-platform (Windows, Linux and MacOS) Java software TCGA2BED from http://bioinf.iasi.cnr.it/tcga2bed/ ("TCGA2BED-v1.0.zip"), that allows to retrieve TCGA data and convert them into the BED, CSV, GTF, JSON, and XML formats.

Executing TCGA2BED

Go to the directory where you extracted the TCGA2BED archive and execute TCGA2BED.jar by double clicking it (for supported operating systems) or by executing the following command from a prompt: `java -jar TCGA2BED.jar`

Start screen

The following main screen of the TCGA2BED software will appear.

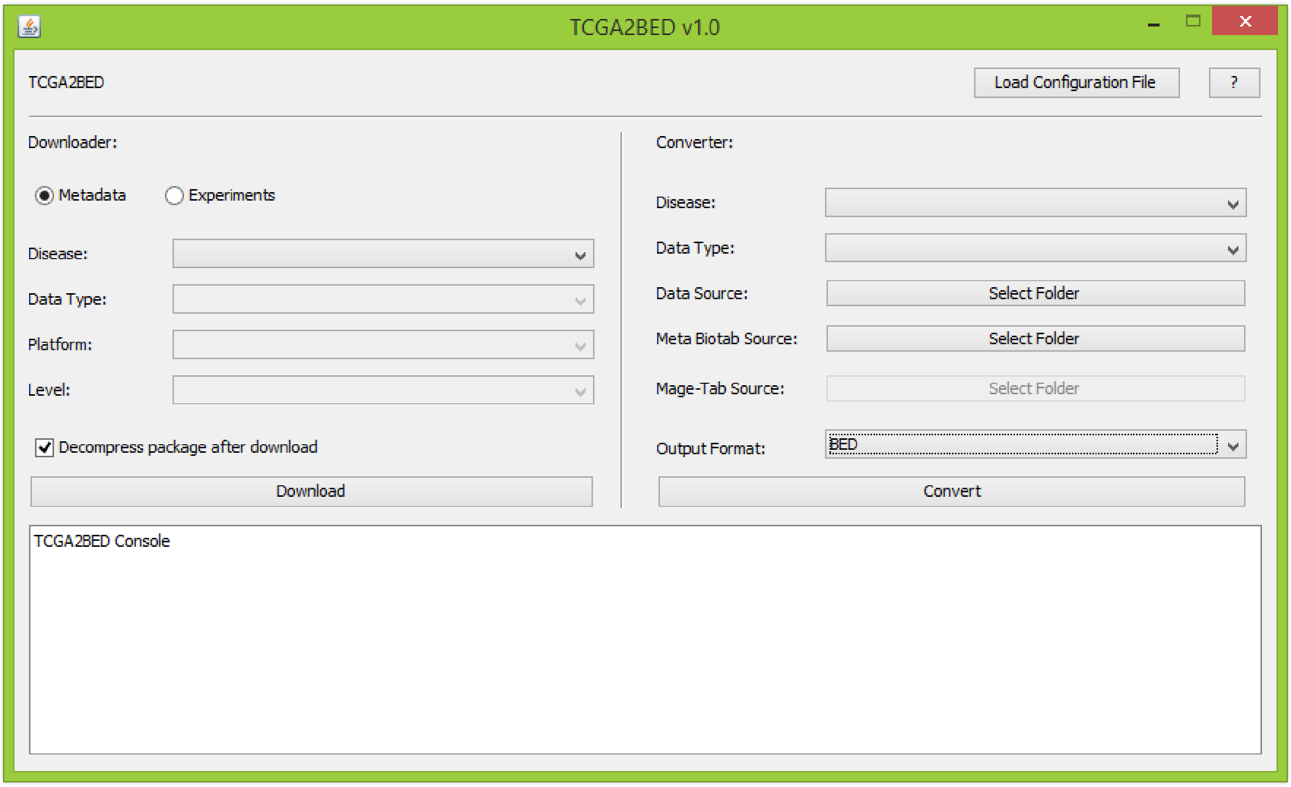

It is composed of two main parts. On the left hand side you can find the *Downloader*, which permits the retrieval of TCGA data. On the right hand side you can find the *Converter*, which allows converting the downloaded data into the BED, CSV, GTF, JSON, and XML formats.

Meta data download

The first step to perform is downloading the meta data (clinical and biospecimen biotab files) for the cancer type you want to analyze. Please select the tumor tag from the drop down menu (*Disease*); a list with the available tumor tags and names is provided at the end of this tutorial. Then press the *Download* button and choose a folder where to save the metadata files.

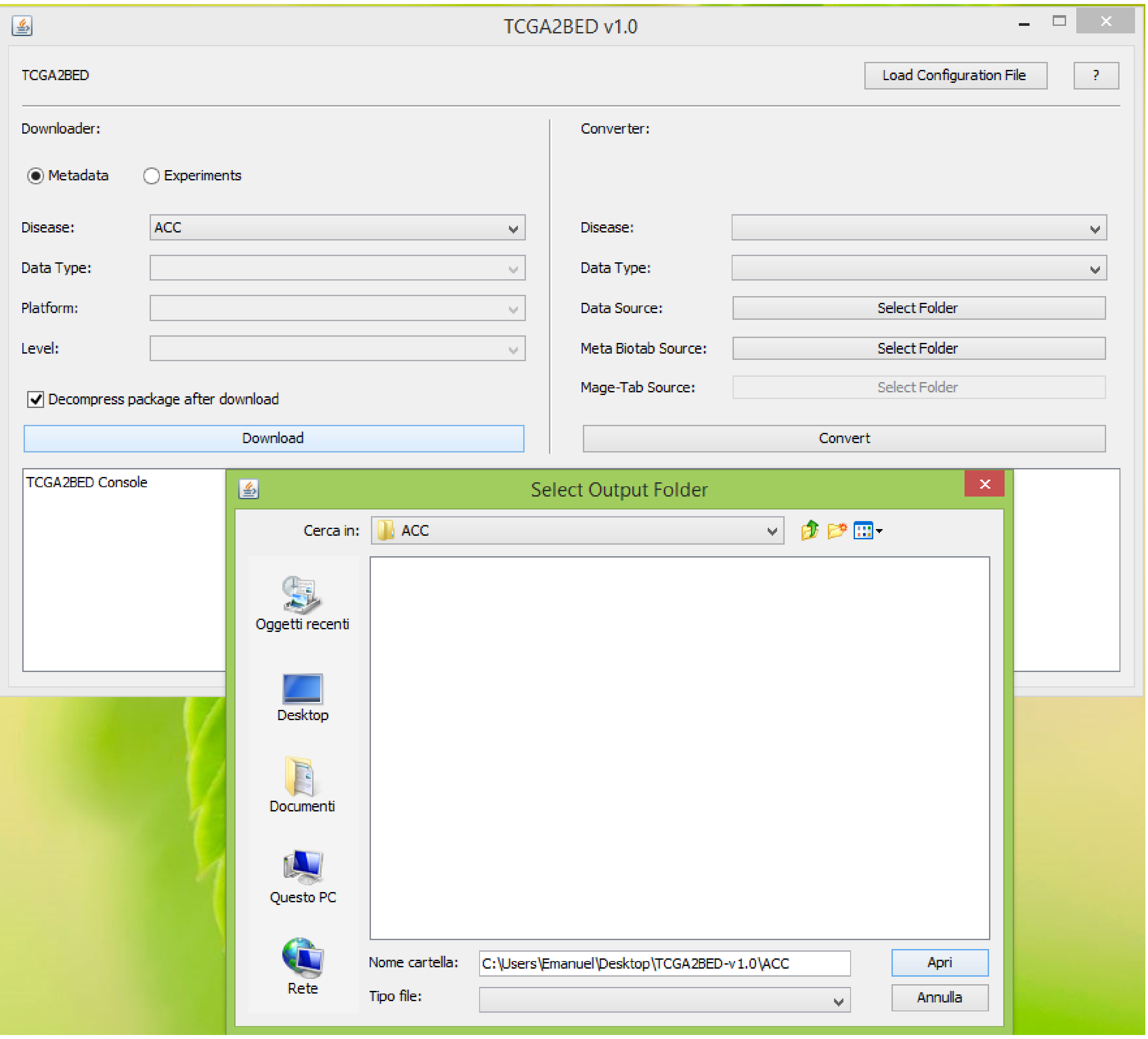

The download will start and you can track the progress from the TCGA2BED console.

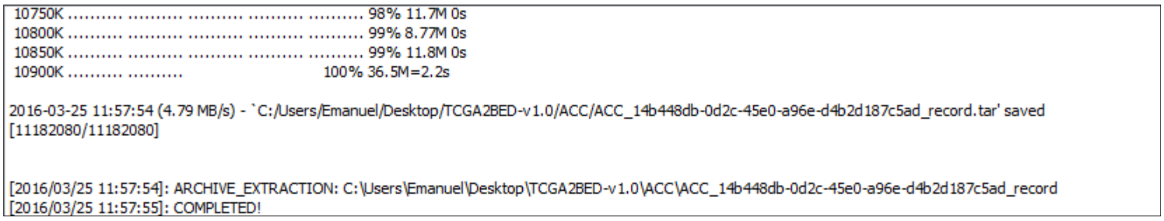

Experimental data download

The second step to perform is downloading the experimental data for the cancer type you want to analyze. Please select the tumor tag from the drop down menu (*Disease*); a list with the available tumor tags and names is provided at the end of this tutorial. Additionally, select the experiment type from the *Data Type* dropdown menu. The available experiment types are: Copy Number Variations (CNV), DNA Methylation, RNA-Seq, RNA-Seq V2, Somatic Mutations (DNA-Seq), miRNASeq. Then press the *Download* button and choose a folder where to save the experimental data files.

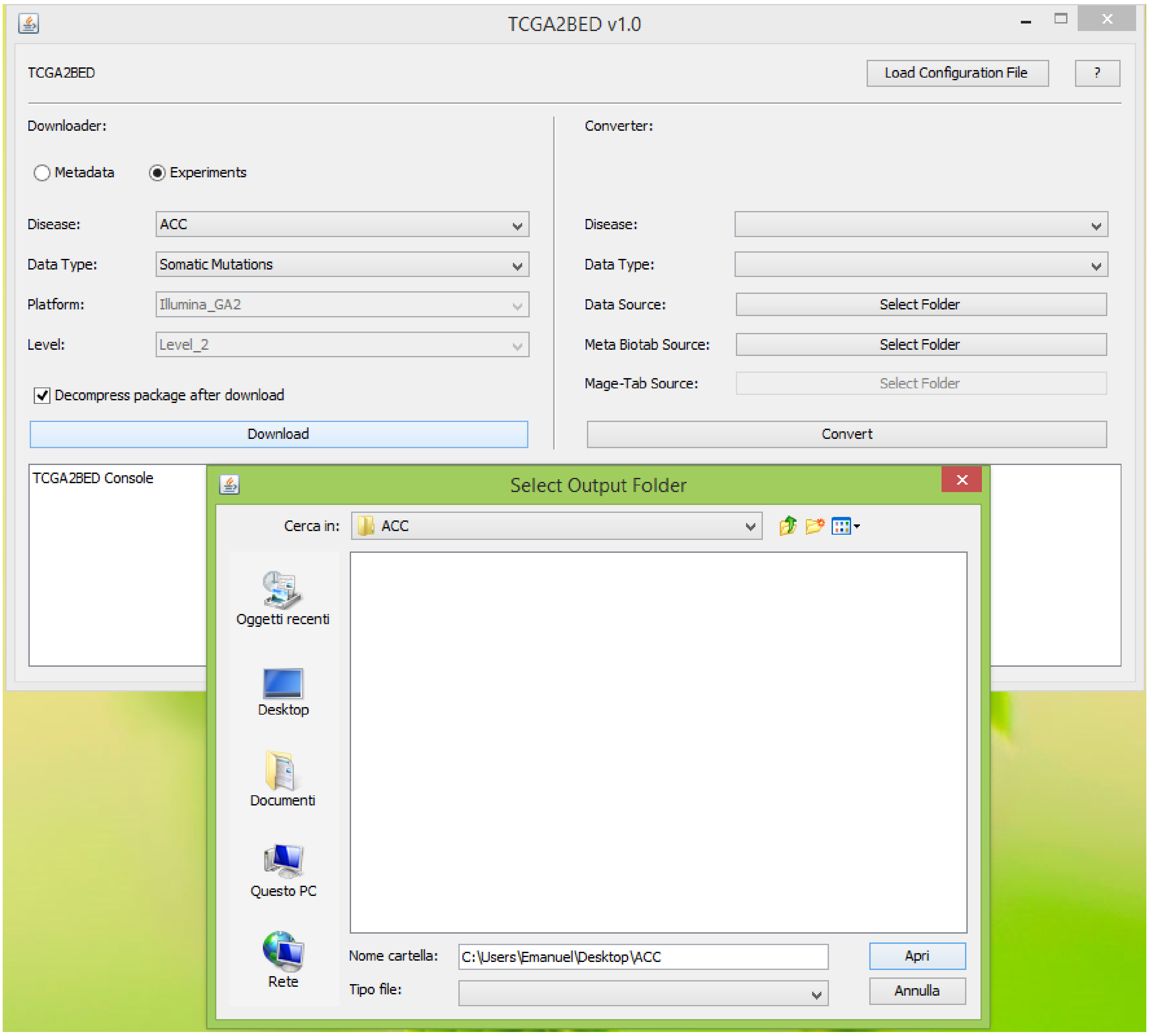

The download will start and you can track the progress from the TCGA2BED console.

```
228100K .......... .......... .......... .......... .......... 99% 2.65M 0s
228150K .......... .......... .......... .......... .......... 99% 1.87M 0s
228200K .......... .......... .......... .......... .......... 99% 2.90M 0s
228250K .......... .......... .......                          100% 2.01M=47s

2016-03-25 12:18:08 (4.76 MB/s) - `C:/Users/Emanuel/Desktop/ACC/broad.mit.edu_ACC.IlluminaGA_DNASeq_curated.Level_2.1.0.0.tar.gz' saved [233755806/23375580

[2016/03/25 12:18:09]: extracting package: broad.mit.edu_ACC.IlluminaGA_DNASeq_curated.Level_2.1.0.0.tar.gz ...
[2016/03/25 12:18:12]: ... extraction completed!
```

Conversion into the supported formats

After the download of the meta data and experimental data, you can start the conversion into the BED, CSV, GTF, JSON, and XML formats (see the "`TCGA2BED_format_definition.pdf`" format definition file that is available as Supplemental material and at http://bioinf.iasi.cnr.it/tcga2bed for further details).

Please select from the drop down menus the *Disease* (through the tumor tag) and the *Data Type* (Copy Number Variations, DNA Methylation, RNA-Seq, RNA-Seq V2, Somatic Mutations, miRNASeq) you want to convert.

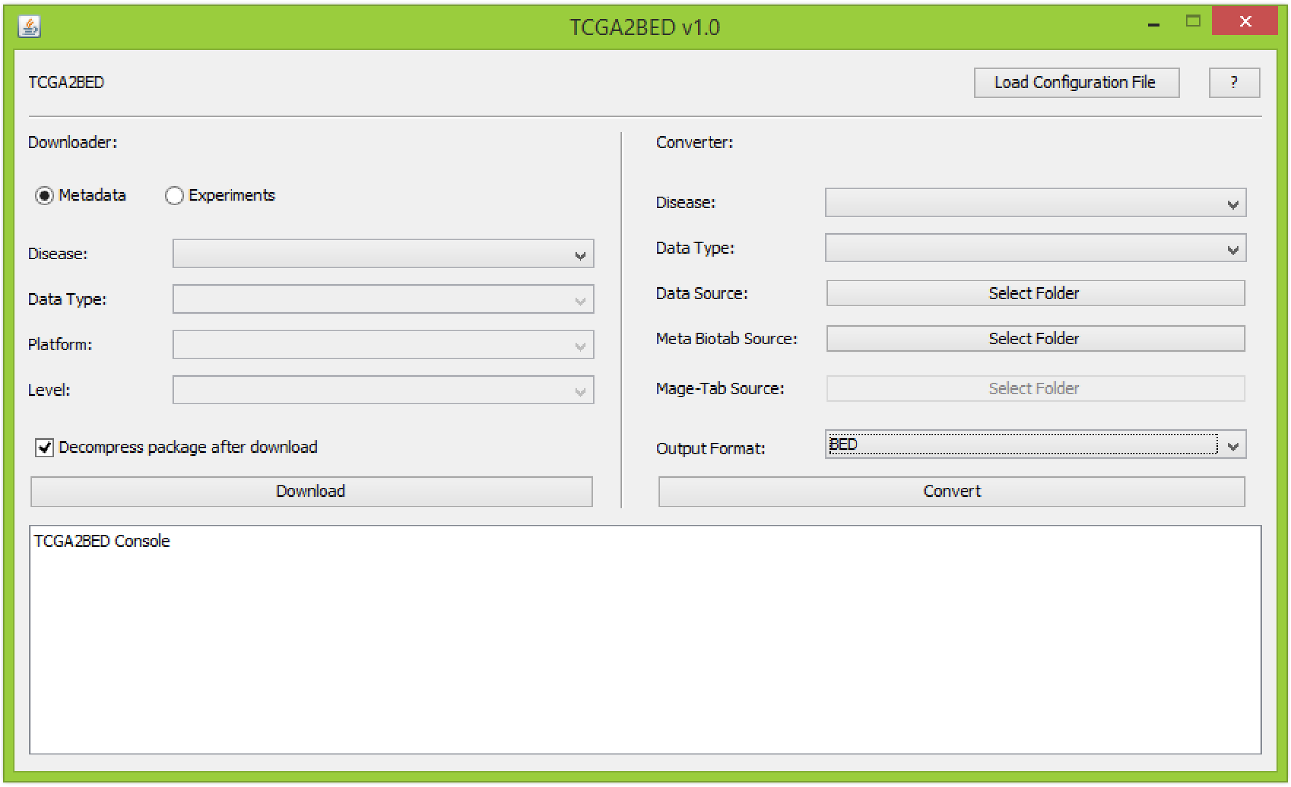

Additionally, specify the folder where you downloaded the experimental data.

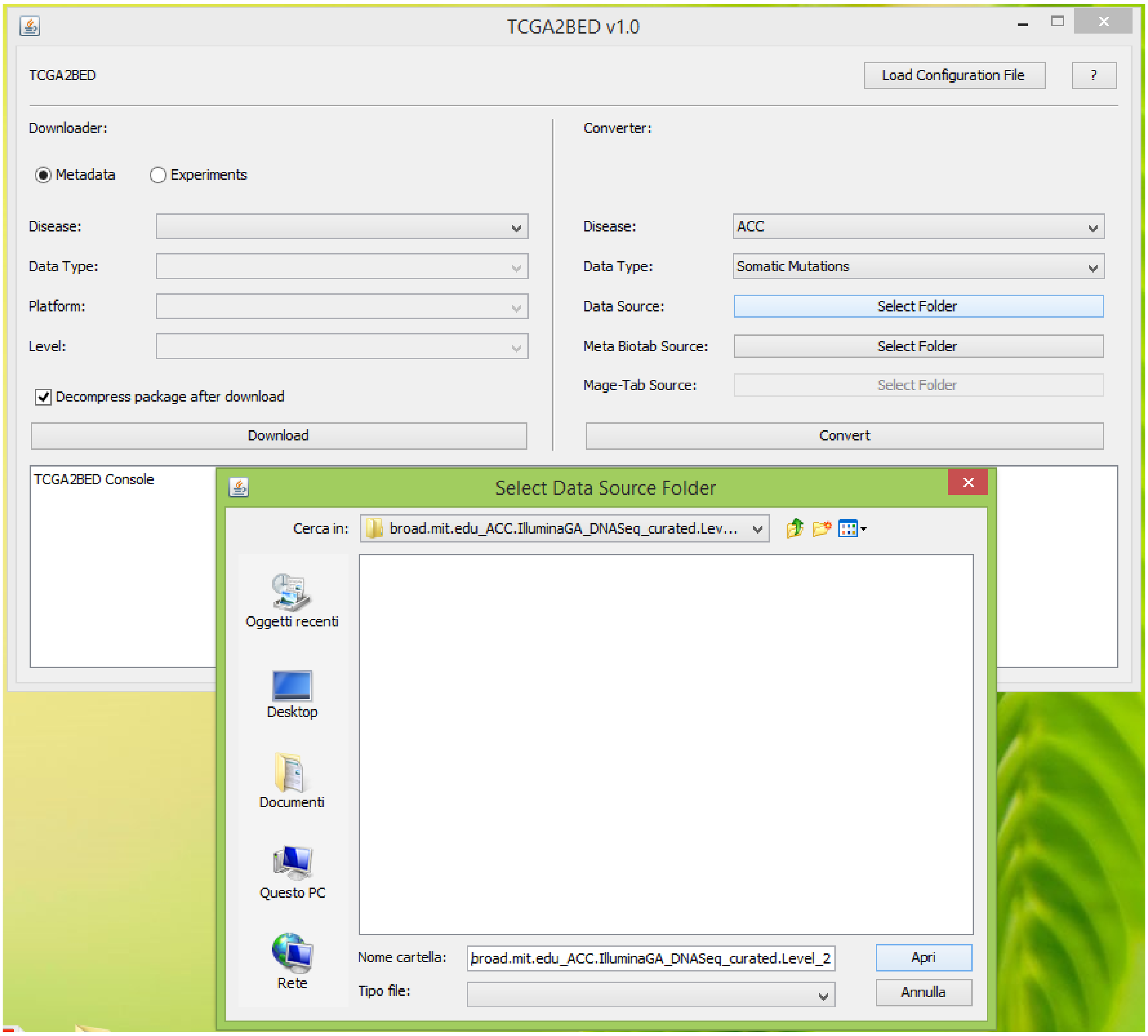

Then, select the folder where you downloaded the metadata biotab files (*Meta Biotab Source*). You have to browse through *Clinical* and *Biotab* in the metadata directory of the extracted archive.

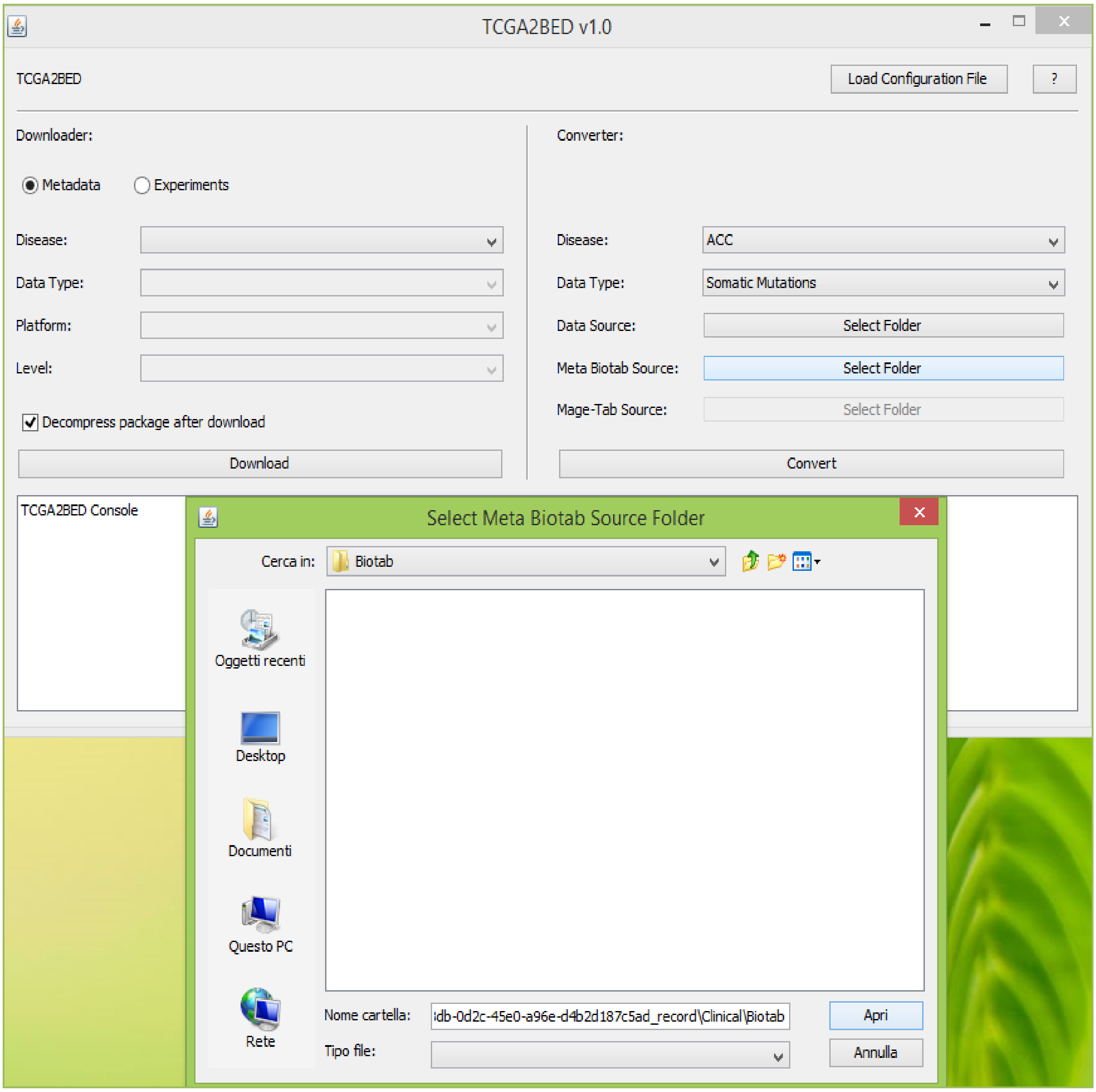

Optionally, if you are converting CNV experiments select the *Mage-Tab Source* directory, which you can find in the root download folder.

Finally, start the conversion by selecting the desired format (BED, CSV, GTF, JSON, or XML), clicking the *Convert* button and choosing the output folder.
You will find the converted files and tab-delimited attribute-value pair meta data files for each experiment in the selected folder.

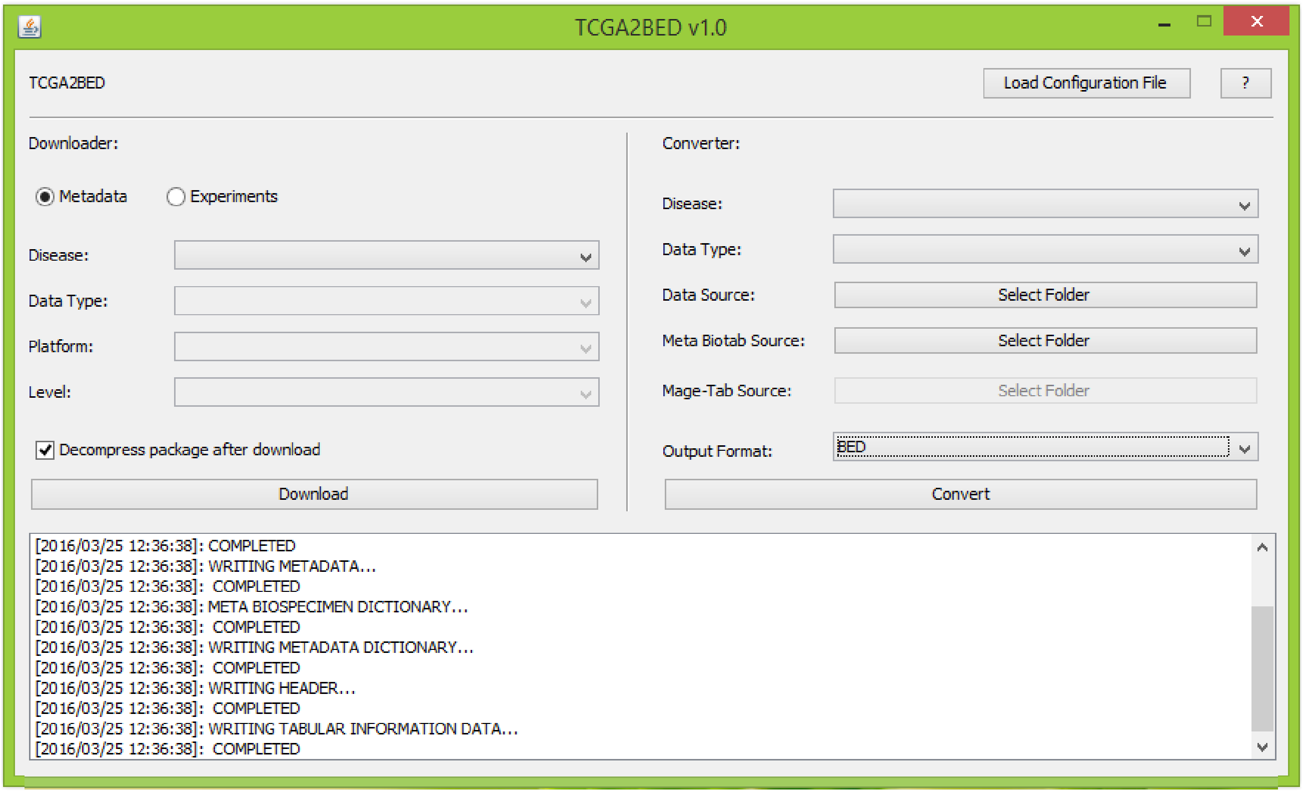

You can start the whole process again with new tumor or experiment types.

Batch download and conversion into the desired format

Through the *Load Configuration File* button you can specify a XML file to download and convert in batch meta data and experimental data of several data types and diseases.

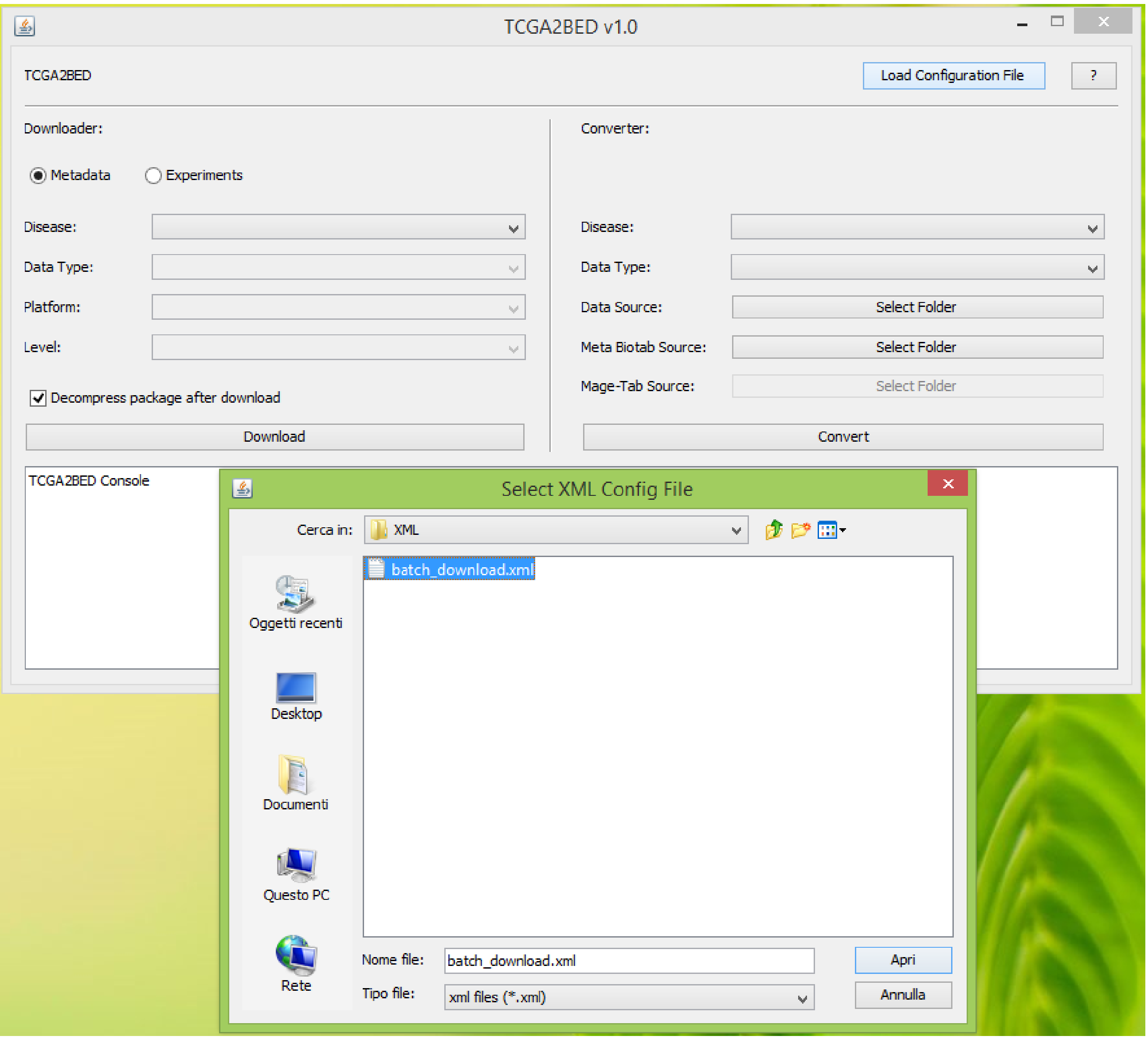

You can find two examples of such xml file in the software package. The first one "`config-download-example.xml`" contains the commands to download meta data and experimental data files from TCGA:

```
<tcga2bed>
	<operation id="0">
		<cmd name="downloadmeta" />
		<attribute name="disease" value="KIRP" />
		<attribute name="output_folder" value="C:/downloads/" />
		<attribute name="autoextract" value="1" />
	</operation>

	<operation id="1">
		<cmd name="downloaddata">
		<attribute name="disease" value="KIRP" />
		<attribute name="data_type" value="DNAMethylation450" />
		<attribute name="output_folder" value="C:/downloads/" />
		<attribute name="autoextract" value="1" />
	</operation>
</tcga2bed>
```

The second one "`config-convert-example.xml`" includes the commands for converting the files into the BED, CSV, GTF, JSON, or XML format:

```
<tcga2bed>
	<operation id="0">
		<cmd name="convert">
		<attribute name="disease" value="KIRP" />
		<attribute name="metadata" value="C:/downloads/KIRP_1dae287c-cc09-
				461e-9488-8d31be3a7325_record/Clinical/Biotab/" />
		<attribute name="additional_metadata" value="null" />
		<attribute name="input_folder" value="C:/downloads/jhu-
			usc.edu_KIRP.HumanMethylation450.Level_3/" />
		<attribute name="output_folder"
			value="C:/downloads/KIRP_DNAMethylation_BED/" />
		<attribute name="data_type" value="DNAMethylation450" />
		<attribute name="data_subtype" value="null" />
		<attribute name="magetab_folder" value="null" />
		<attribute name="output_format" value="bed" />
	</operation>
</tcga2bed>
```

For creating your own XML file, please follow following directions.

Each operation is defined as a XML block denoted by the *operation* tag followed by an incremental numeric identifier needed to preserve the execution sequence.

Two types of XML tags are required to define an operation:

1. *cmd* tag followed by the name of the current operation that can be one of the following commands:
    a. *downloadmeta* to download clinical data about a specific tumor;
    b. *downloaddata* to download a particular type of experiments about a tumor;
    c. *convert* to convert experiments from the TCGA format into the BED format.

2. The following attribute tags are required to configure an operation depending on the previously selected command. Please follow the example files to use them properly.
    a. *disease* denotes a specific tumor tag (all tags are listed at the end of this document);
    b. *metadata* contains the full path to the clinical data in biotab format;

c. *additional_metadata* is a field and contains the full path to a file with user defined clinical data, if not present please set it to *null*;
d. *input_folder* is the full path to the folder that contains experiments in TCGA format;
e. *output_folder* is the output directory where the converted BED files will be generated;
f. *data_type* denotes the type of the experiments contained in the folder specified in the *input_folder* field, and could be *DNAMethylation27, DNAMethylation450, DNASeq, RNASeq, RNASeqV2, miRNASeq,* and *CNV*;
g. *data_subtype* is required for *DNA Methylation*, *RNASeq*, *RNASeqV2,* and *miRNASeq* only. The allowed values for this field are, *gene, exon,* and *spljxn* for *RNASeq*, *gene, exon, spljxn*, and *isoform* for *RNASeqV2*, and *mirna*, and *isoform* for *miRNASeq*; if you are not converting these experiments please set it to *null*;
h. *magetab_folder* is required for the conversion of *CNV* experiments only and contains the full path to the folder with magetab data; if you are not converting *CNV* please set it to *null*;
i. *autoextract* is a binary parameter (*0* or *1*), if set to *1* the data will be automatically decompressed after the download process;
j. *output_format* is an optional parameter that can be set to BED, CSV, GTF, JSON, or XML to define the desired output format.

Data repository

The ftp site ftp://bioinf.iasi.cnr.it contains an up-to-date archive with the experimental and meta data from TCGA converted into the BED format.

Citation

If you use TCGA2BED please cite "*TCGA2BED: extracting, extending, integrating, and querying The Cancer Genome Atlas by Fabio Cumbo, Giulia Fiscon, Stefano Ceri, Marco Masseroli and Emanuel Weitschek*".

Contacts

For comments and questions please contact Fabio Cumbo (fabio.cumbo@iasi.cnr.it) or Emanuel Weitschek (emanuel.weitschek@iasi.cnr.it).

Appendix: tumor tags and tumor names

ACC	Adrenocortical carcinoma
BLCA	Bladder Utothelial Carcinoma
BRCA	Breast Invasive Carcinoma
CESC	Cervical squamous cell carcinoma and endocervical adenocarcinoma
CHOL	Cholangiocarcinoma
COAD	Colon adenocarcinoma
DLBC	Lymphoid Neoplasm Diffuse Large B-cell Lymphoma
ESCA	Esophageal carcinoma
GBM	Glioblastoma multiforme
HNSC	Head and Neck squamous cell carcinoma
KICH	Kidney Chromophobe
KIRC	Kidney renal clear cell carcinoma
KIRP	Kidney renal papillary cell carcinoma
LAML	Acute Myeloid Leukemia
LGG	Brain Lower Grade Glioma
LIHC	Liver hepatocellular carcinoma
LUAD	Lung adenocarcinoma
LUSC	Lung squamous cell carcinoma
MESO	Mesothelioma
OV	Ovarian serous cystadenocarcinoma
PAAD	Pancreatic adenocarcinoma
PCPG	Pheochromocytoma and Paraganglioma
PRAD	Prostate adenocarcinoma
READ	Rectum adenocarcinoma
SARC	Sarcoma
SKCM	Skin Cutaneous Melanoma
STAD	Stomach adenocarcinoma
TGCT	Testicular Germ Cell Tumors
THCA	Thyroid carcinoma
THYM	Thymoma
UCEC	Uterine Corpus Endometrial Carcinoma
UCS	Uterine Carcinosarcoma
UVM	Uveal Melanoma
